# Synthesis of 2-Deoxybrassinosteroids Analogs with 24-nor, 22(*S*)-23-Dihydroxy-Type Side Chains from Hyodeoxycholic Acid

**DOI:** 10.3390/molecules23061306

**Published:** 2018-05-29

**Authors:** Rodrigo Carvajal, Cesar González, Andrés F. Olea, Mauricio Fuentealba, Luis Espinoza

**Affiliations:** 1 Departamento de Química, Universidad Técnica Federico Santa María, Avenida España 1680, Valparaíso 2340000, Chile; rodrigo.carvajal@postgrado.usm.cl (R.C.); cesar.gonzalez@usm.cl (C.G.); 2 Instituto de Ciencias Químicas Aplicadas, Facultad de Ingeniería, Universidad Autónoma de Chile, Santiago 8910339, Chile; 3 Laboratorio de Cristalografía, Pontificia Universidad Católica de Valparaíso, Avenida Universidad 330, Curauma, Valparaíso 2340000, Chile; mauricio.fuentealba@pucv.cl

**Keywords:** brassinosteroid analogs, hyodeoxycholic acid, 2-deoxybrassinosteroids, synthesis, short side chain

## Abstract

Natural brassinosteroids are widespread in the plant kingdom and it is known that they play an important role in regulating plant growth. In this study, two new brassinosteroid analogs with shorter side chains but keeping the diol function were synthesized. Thus, the synthesis of 2-deoxybrassinosteroids analogs of the 3α-hydroxy-24-nor, 22,23-dihydroxy-5α-cholestane side chain type is described. The starting material is a derivative from hyodeoxycholic acid (**4**), which was obtained with an overall yield of 59% following a previously reported five step route. The side chain of this intermediate was modified by oxidative decarboxylation to get a terminal olefin at the C22-C23 position (compound **20**) and subsequent dihydroxylation of the olefin. The resulting epimeric mixture of **21a**, **21b** was separated and the absolute configuration at the C22 carbon for the main product **21a** was elucidated by single crystal X-ray diffraction analysis of the benzoylated derivative **22**. Finally, lactonization of **21a** through a Baeyer-Villiger oxidation of triacetylated derivative **23**, using CF_3_CO_3_H/CHCl_3_ as oxidant system, leads to lactones **24** and **25** in 35% and 14% yields, respectively. Deacetylation of these compounds leads to 2-deoxybrassinosteroids **18** and **19** in 86% and 81% yields. Full structural characterization of all synthesized compounds was achieved using their 1D, 2D NMR, and HRMS data.

## 1. Introduction

**Figure 1:**
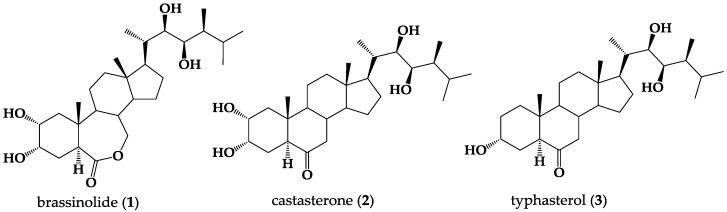
Structure of brassinolide (**1**), castasterone (**2**) and typhasterol (**3**).

Since the discovery of brassinolide (**1**), a polyhydroxysteroidal hormone that regulates plant growth and development, other brassinosteroids (BRs) have been found throughout all the plant kingdom and much effort has been dedicated to the synthesis of BR analogs. Most of this work has been focused on determining the structural requirements that these compounds should possess to elicit strong biological activity [1,2,3]. For example, in Figure 1 are shown the chemical structures of **1**, castasterone (**2**) and typhasterol (**3**). The latter is a natural 2-deoxybrassinosteroid that may act as important biosynthetic precursors of more active brassinosteroids [4,5,6,7].

**Figure 2:**
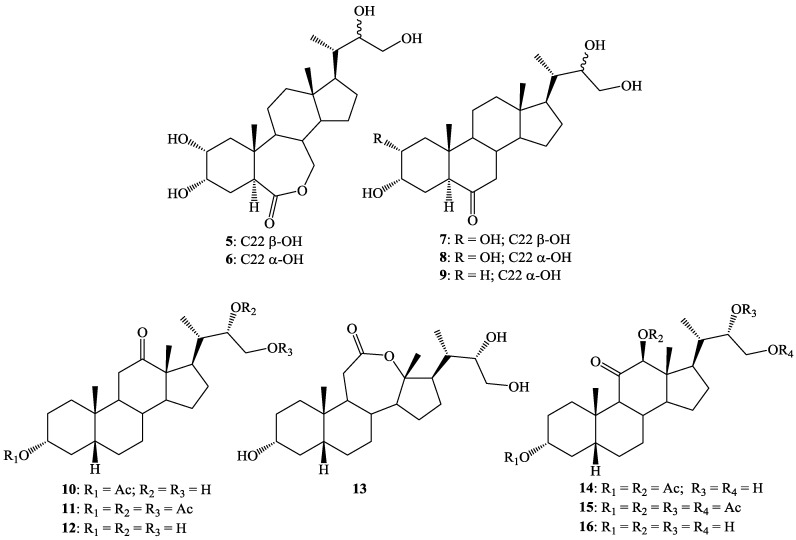
Synthetic 24-nor-22(*S*),23-dihydroxy analogs **5**–**16**.

Natural occurring BRs show a variety of structural modifications in the A/B ring, but it seems that a vicinal 22*R*,23*R* diol structural functionality in the side chain is essential for high biological activity. In recent decades, many BR analogs with structural changes on the A/B rings and/or on the side chain (shorter side chains, different oxygenated functions, spirostanic, aromatic and cyclic substituents, methyl esters, carboxylic acids) have been synthesized [8,9,10,11,12]. Surprisingly, some BR analogs with drastic structural modifications in the side chain have also shown interesting activities as plant growth regulators. Thus, the structural requirement of a side chain with a *cis* C-22, C-23-diol, preferentially with *R*, *R* configurations, and a C-24 methyl or ethyl substituent, seems to be contradicted by an important number of BR analogs exhibiting strong biological activities. For example, in Figure 2 are shown a series of BR analogs with 24-nor-22,23-dihydroxy-type side chains, i.e., BRs analogs with shorter side chain as compared to naturally occurring BRs. 

Hyodeoxycholic acid (**4**) has been used in the synthesis of several BRs analogs because its structure is similar to that of active BRs and it is commercially available. Thus, compounds **5**–**8** and **8**–**9** have been synthesized from **4** following different synthetic routes [13,14]. In both cases, the modification in the side chain was achieved by decarboxylation and subsequent dihydroxylation of a terminal olefin. From these compounds only **8** and **9** were evaluated as potential neuroinflammation inhibitors [14]. On the other hand, compounds **10**–**13** were synthesized from deoxycholic acid, bearing oxygenated functions in ring C, with 24-nor-22(*S*),23-dihydroxy side chain and cis A/B ring fusion [15], whereas analogues **14**–**16** were obtained from deoxycholic acid with 11-oxo-functionalized on C ring, 24-nor-22(*S*),23-dihydroxy and 22(*S*),23-diacetoxy [16]. Interestingly, compounds **10** and **13** have shown growth promoting activity in hypocotile elongation and cothyledon expansion in a radish bioassay [17].

**Figure 3:**
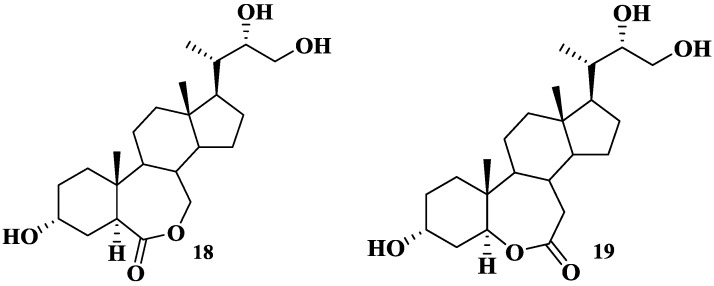
New 2-deoxybrassinosteroids analogs (**18** and **19**) with 3α-hydroxy-24-nor-22,23-dihydroxy-5α-cholestane side chain type.

From the synthetic point of view, the tremendous effort dedicated to obtain a number of synthetic analogs has led to development of some convenient, effective and general methods of synthesis applicable to this compound class [18,19,20,21,22,23,24,25,26,27,28,29,30,31]. In this work, we describe the synthesis of new 2-deoxybrassinosteroid analogs bearing a shorter side chain but retaining the diol function, i.e., a 3α-hydroxy-24-nor-22,23-dihydroxy-5α-cholestane side chain type. The starting material is **17**, a hyodeoxycholic acid derivative. Following this procedure two new 2-deoxybrassinosteroids analogs (compounds **18** and **19**, Figure 3) have been prepared. The full structural characterization of these analogs is also given.

## 2. Results

**Scheme 1:**
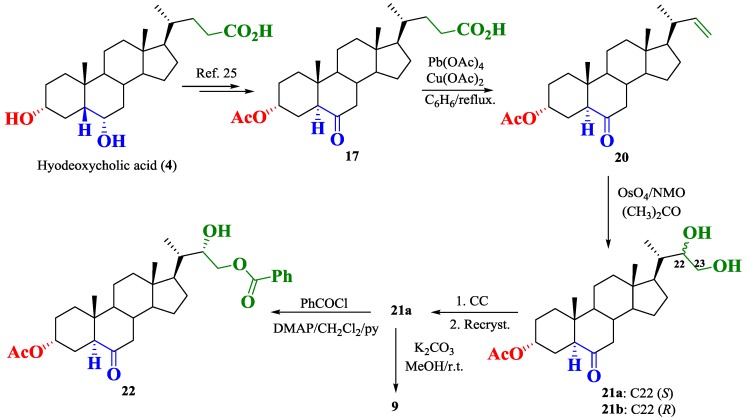
Synthesis of compound **21a**, followed by selective benzoylation at the C-23 position to obtain the derivative **22**, and synthesis of brassinosteroid analog **9**. CC stands for Column Chromatography.

The main goal of this work was to synthesize BR analogs where the main structural change was a reduction of the side alkyl chain length, as compared to brassinolide (**1**), but keeping the glycol function at the C22−C23 position. In the steroidal nucleus, the introduction of a glycol function at the C22−C23 position via dihydroxylation with OsO_4_ requires the presence of a terminal double bond. In the case of hyodeoxycholic acid (**4**) this can be accomplished by oxidative decarboxylation using a Pb(OAc)_4_/Cu(OAc)_2_ system. This method has been proposed to obtain terminal double bonds from carboxylic acids [32], and has been used for the degradation of bile acid side chains [33], synthesis of BR analogs [14,15,17], and specifically for decarboxylation of the side chain of hyodeoxycholic acid (**4**) and derivatives [34,35]. Alternatively, the carboxylic degradation reaction may be carried out using PhI(OAc)_2_/CuSO_4_ system [13,34,35,36,37,38,39,40,41,42,43,44,45]. Hyodeoxycholic acid (**4**) is a common starting material because it is easily available, and it has been previously used to synthesize a number of BR analogs. Related to this work, we have recently reported the synthesis of compound **17** in a five step route with an overall yield of 59% [46]. This compound will be the intermediate for the synthesis of **18** and **19** (Scheme 1).

### 2.1. Synthesis of Brassinosteroids Analogs

Oxidative decarboxylation of the side chain of compound **17**, with the Pb(OAc)_4_/Cu(OAc)_2_ system, leads to olefin **20** in 75% yield. Formation of **20** was confirmed by ^1^H-NMR and ^13^C-NMR. 

Dihydroxylation of alkene **20** with OsO_4_ produces an epimeric mixture of **21a** and **21b** in 72% yield (Scheme 1). This is an expected outcome for this reaction in steroidal nucleus with a terminal double bond at the C22−C23 position (24-nor-chol-22-ene), and the 22(*S*) alcohol is stereoselectively obtained [47]. Thus, epimeric mixtures have been obtained during the preparation of analogs **10**, **11** and **12** (Figure 2) [15,16,17].

Integration areas of signals in the ^1^H-NMR spectrum of mixture **21a**/**21b**, appearing at δ_H_ = 0.965 and 0.928 ppm, respectively, and assigned to the H-21 methyl hydrogen (CH_3_-C20), indicates that the major component on this mixture is the less polar glycol **21a** in a ratio 7.5:1.0. Recrystallization of a mixture of **21a**/**21b** (MeOH/Et_2_O = 3/1) allowed for isolation of **21a** in 64.0% yield.

The stereochemistry at the C22 carbon for compound **21a** was assumed to be 22(*S*) based on previous results reported for similar hydroxylation reactions used to obtain analogues **10**, **13** and **14** (Figure 2) [15,16,17]. In order to establish the absolute configuration at C22 carbon for compound **21a**, the benzoylated derivative **22** (Scheme 1) was prepared. Treatment of **21a** with PhCOCl/DMAP in CH_2_Cl_2_ and pyridine led to selective esterification of C23 as the only reaction product in 94.0% yield. 

**Figure 4:**
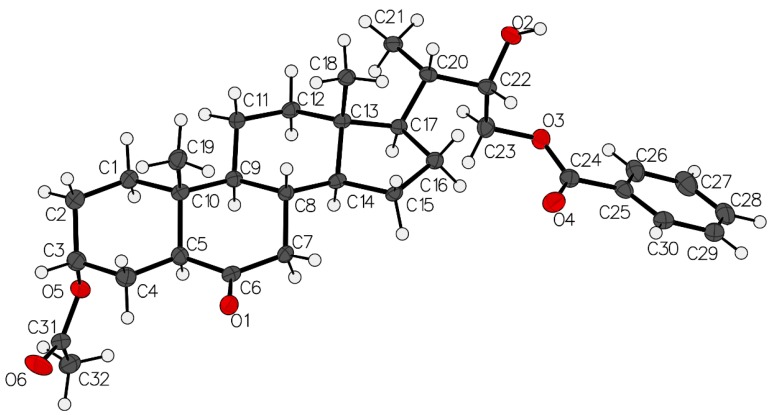
ORTEP diagram of derivative **22** showing full atom-numbering scheme. Thermal ellipsoids are drawn at the 50% probability level.

Finally, the molecular and crystalline structure of derivative **22** was determined using single crystal X-ray diffraction techniques. This structure crystallizes in the orthorhombic Sohncke space group *P*2_1_2_1_2_1_. The ORTEP diagram appears in Figure 4, whereas X-ray data, bond distances and angles are given in Tables S1–S3, respectively, of the Supplementary Material.

The absolute configuration ***S*** for C22 of compound **22** cannot be reliably determined using only the Flack’s parameter value of −0.2(3), calculated by using 1169 quotients of the type [(*I*^+^) − (*I*^−^)]/[(*I*^+^) + (*I*^−^)] [48]. Nevertheless, the analysis of Bayesian statistics of Bijovet pairs it is a much simpler and reliable method to determine the absolute configuration for molecules that contain atoms no heavier than oxygen [49]. The resulting values for the analysis of 2386 Bijovet pairs, Hooft’s parameter y: 0.0(2); P2(true): 1.000; P2(false): 1.743 × 10^−6^; P3(true): 0.973; P3(false): 1.695 × 10^−6^ and; P3(racemic twin): 0.027, have confirmed the absolute structure for compound **22**. Additionally, considering that compound **22** was synthesized using enantiopure precursors, the configurations *R*, *S*, *S*, *S*, *R*, *S*, *S*, *R* and *S* have also been verified for atoms C3, C5, C8, C9, C10, C13, C14, C17 and C20, respectively.

**Scheme 2:**
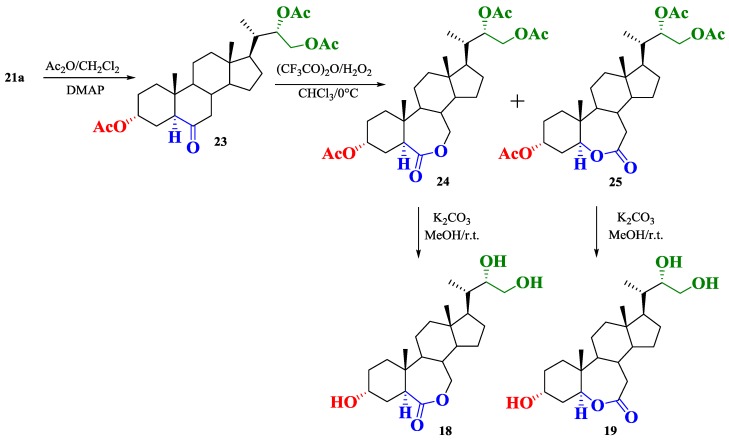
Synthesis of triacetylated derivative **23** and its subsequent Baeyer-Villiger oxidation to obtain new 2-deoxybrassinosteroids **18** and **19**.

A mild saponification reaction (K_2_CO_3_/MeOH, r.t.) of glycol **21a** gave the brassinosteroid analog **9** in 97% yield (Scheme 2). This compound has been previously obtained by using a different synthetic route, and its structure was determined by ^1^H-, ^13^C-NMR spectroscopy, EIMS and HRMS spectrometry. However, as the assignment of NMR signals was not performed [14] both the ^1^H- and ^13^C-NMR spectra of this compound are given in the Supplementary Material. 

In order to obtain 2-deoxybrassinosteroid analogs **18** and **19** a lactone group (B-homo-7-oxa and B-homo-6-oxa) must be introduced in the B ring of **21a** (Scheme 2). It is known that Baeyer-Villiger oxidation of 5α-6-keto-steroids with oxygenated substituents at 2α−3α and 3α positions occurs with regioselective control, favoring 7-oxalactone formation, when electron-withdrawing substituents (acetyl [13,35,36,37,43,50], benzoyl [36,50], tosyl [36,50], trifluoroacetyl [50] and acetonide [42] groups) are present in the C-3 position [50]. Also, the use of CF_3_CO_3_H as the oxidant agent has a marked effect upon the 6-oxa/7-oxa ratio, and can lead to preferential formation of the desired 7-oxa isomer [50]. Additionally, lactonization global yields are greater than those obtained when there are hydroxyl groups in the steroid structure [45]. For these reasons, Baeyer-Villiger oxidation of triacetylated derivative **23** instead of **21a** is performed with CF_3_CO_3_H/CHCl_3_ as the oxidant. Similar regioselectivity has been observed in the oxidation of a series of sterols with *m*-CPBA/NaHCO_3_/CH_2_Cl_2_ system, but the rates of these reactions are very slow [50].

Standard acetylation (Ac_2_O/DMAP) of compound **21a** (Scheme 2) leads to triacetylated derivative **23** with 97% yield. 

Baeyer-Villiger oxidation of **23** with CF_3_CO_3_H/CHCl_3_ system produces lactones **24** and **25** with 35% and 14% yields, respectively. Finally, deacetylation reaction of lactones **24** and **25** under mild conditions (K_2_CO_3_/MeOH, at room temperature) produced the new analogs of 2-deoxy-brassinosteroids **18** and **19** with 86% and 81% yields, respectively.

### 2.2. Elucidation of Compound Structures

The full structure assignment of compounds **20**, **21a**, **9**, **22**, and **23** were carried out by analysis of spectroscopic data obtained from ^1^H-NMR, ^13^C-NMR, and HRMS of pure and isolated compounds.

In the ^1^H-NMR of compound **20** the protons H-22, H*_trans_*-23 and H*_cis_*-23 appear at δ_H_ = 5.66 ppm (H-22), 4.93 ppm (H*_trans_*-23) and 4.83 ppm (H*_cis_*-23). In the ^13^C-NMR the carbons C22 and C23 appear at δ_C_ = 144.89 and 111.84 ppm, respectively (Table 1). These data were consistent with those reported for a similar structure but with hydroxyl function at C-3α instead of acetyl group [14,43].

In the ^1^H-NMR of **21a** signals appearing at chemical shifts δ_H_ = 3.79 ppm, 3.64 ppm, and 3.51 ppm are assigned to protons H-22, H-23a and H-23b, respectively. On the other hand, in the ^13^C-NMR spectrum, the signals at δ_C_ = 73.69 and 62.22 ppm correspond to carbinolic carbons C22 and C23, respectively (see Table 1). 

The ^1^H-NMR of triol **9** shows signals at δ_H_ = 4.04–4.03, 3.71, 3.60, and 3.40 ppm, which are assigned to hydrogens H-3, H-22, H-23a and H-23b, respectively. In the ^13^C-NMR three carbinolic carbons (C22, C-3 and C23) were observed at δ_C_ = 75.19, 66.02 and 63.19 ppm, (Table 1).

The structure of derivative **22** was established mainly by 1D and 2D NMR spectroscopy. In the ^1^H-NMR spectrum the aromatic protons appear at δ_H_ = 8.05 (H*_Ar_*-2’), 7.58 (H*_Ar_*-4’), and 7.46 (H*_Ar_*-3’) ppm. Additionally, two low field shifted signals at δ_H_ = 4.50 (dd, *J* = 11.4 and 1.7 Hz, 1H) and 4.20 (dd, *J* = 11.3 and 4.2 Hz, 1H) ppm correspond to H-23a and H-23b. In the ^13^C-NMR spectrum the signal at δ_C_ = 167.01 ppm is assigned to carbonyl of aromatic ester, whereas the signals at δ_C_ = 129.86, 129.63, 128.45 and 133.22 ppm (Table 1) are assigned to aromatic ring (each one of signals at δ_C_ = 129.63 and 128.45 ppm correspond to two symmetrical carbons of the aromatic ring). In the 2D HMBC spectrum a heteronuclear correlation at ^3^*J*_H-C_ between H-23a (δ_H_ = 4.50 ppm) with carbonyl of aromatic ester (δ_C_ = 167.01 ppm) was observed, confirming the presence of benzoyl ester at C23 position. The structure of compounds **18**, **19**, **24** and **25** were mainly elucidated by analysis of data obtained from ^1^H, ^13^C, ^13^C DEPT-135, 2D HSQC, 2D HMBC NMR, and HRMS measurements. 

**Figure 5:**
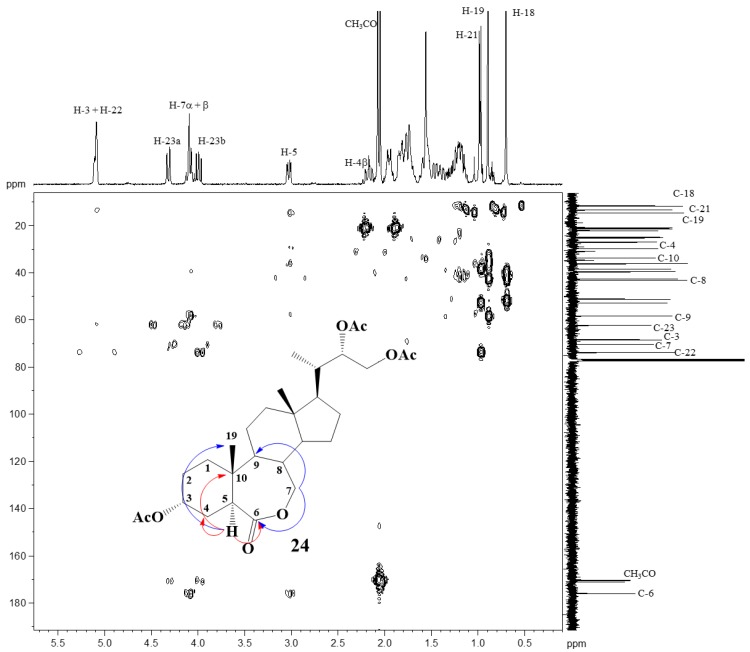
Inverse detection heteronuclear-correlated 2D ^1^H-^13^C HMBC contour plot, and main ^2^*J*_HC_ (red) and ^3^*J*_HC_ (blue) correlations observed for hydrogens H-5α and H-7 of compound **24** (7-oxalactone).

For compound **24**, the position of the 7-oxa lactone function was established from the ^1^H-NMR spectrum where a signal observed at δ_H_ = 4.13–4.04 ppm (m, 2H), was assigned to hydrogens H-7 and correlated by 2D ^1^H-^13^C HSQC with the signal at δ_C_ = 70.31 ppm (CH_2_-7 from ^13^C and ^13^C DEPT-135 spectra, Table 2). Additionally, important heteronuclear correlations were obtained for hydrogens H-5α and H-7 from a 2D ^1^H-^13^C HMBC spectrum, i.e., (i) H-7 shows ^3^*J*_HC_ correlations with the signal at δ_C_ = 175.97 ppm (assigned to carbon C-6, C=O of lactone function, Table 2), and signals at δ_C_ = 58.31 ppm (assigned to carbon C-9); and ^2^*J*_HC_ correlation with signal appearing at δ_C_ = 39.42 ppm (assigned to carbon C-8); (ii) H-5α at δ_H_ = 3.03 ppm shows ^3^*J*_HC_ correlation with signals at δ_C_ = 14.54, and 58.31 ppm, which were assigned to carbons CH_3_-19 and C-9, respectively (Table 2); (iii) H-5α exhibits ^2^*J*_HC_ correlation with signals at δ_C_ = 29.7, 36.1 and 176.0 ppm, assigned to carbons C-4, C-10 and C-6, respectively. These correlations are depicted in the 2D HMBC spectrum shown in Figure 5. These observations confirmed unequivocally the 7-oxalactone position for compound **24**. 

**Figure 6:**
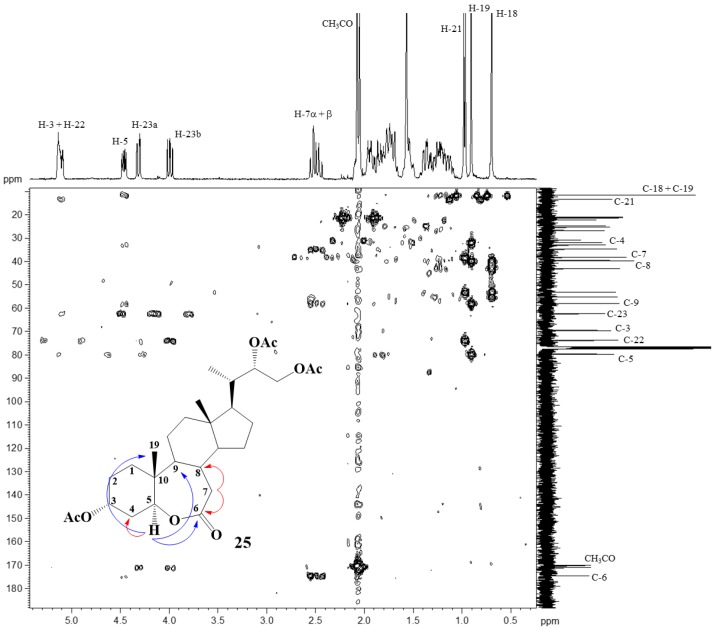
Inverse Detection Heteronuclear-Correlated 2D ^1^H-^13^C HMBC Contour Plot, and Major ^2^*J*_HC_ (red) and ^3^*J*_HC_ (blue) Correlations Observed for Protons H-5α and H-7 of Compound **25** (6-oxalactone).

A similar analysis was performed to determine the structure of 6-oxalactone **25**. Thus, in the ^1^H-NMR spectrum a signal at δ_H_ = 4.46 ppm was assigned to H-5α, and correlated by 2D ^1^H-^13^C HSQC with the signal at δ_C_ = 79.68 ppm (CH with impair multiplicity from DEPT-135 spectrum). Additionally, H-5α shows ^2^*J*_HC_ correlation with signal at δ_C_ = 32.95 ppm that is assigned to carbon C-4; and ^3^*J*_HC_ correlation with signals at δ_C_ = 11.59, 57.94 and 174.64 ppm, which are assigned to carbons CH_3_-19, C-9 and C-6, respectively (C=O, of lactone function, Table 2). On the other hand, the ^1^H-NMR signal at δ_H_ = 2.55–2.43 ppm, corresponding to H-7 (2H, m), shows ^2^*J*_HC_ correlation with signals appearing at δ_C_ = 38.31 and 174.64 ppm, which were assigned to carbons C-8 and C-6, respectively (Table 2); and ^3^*J*_HC_ correlation with signals at δ_C_ = 57.97 ppm, assigned to carbons C-9. These correlations are shown in the 2D HMBC spectrum in Figure 6.

**Figure 7:**
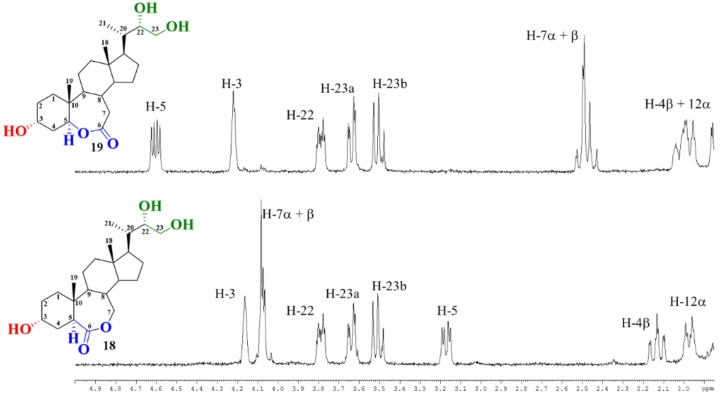
Major differences between chemical shift of hydrogens H-5 and H-7. Partial ^1^H-NMR spectra (1.85–5.00 ppm) of lactones 2-Deoxybrassinosteroids **18** (**bottom**) and **19** (**top**).

Similar analyses were performed to determine the structure of compounds **18** and **19**. In Figure 7 are shown parts of ^1^H-NMR spectra of compounds **18** and **19** where the major differences in chemical shift of protons H-5 and H-7 are observed for both molecules. For example, H-5 in compound **18** (7-oxalactone) appears at higher field (δ_H_ = 3.17 ppm) than in compound **19** (6-oxalactone) (δ_H_ = 4.60 ppm) (Figure 7). Similarly, H-7α and β in compound **18** are observed at downfield (δ_H_ = 4.08–4.07, m, 2H), while in 6-oxalactone **19** these H-atoms are displaced to high field (δ_H_ = 2.53–2.43, m, 2H) (Figure 7).

## 3. Materials and Methods

### 3.1. General Experimental Methods

All reagents were purchased from commercial suppliers, and used without further purification. Melting points were measured on a SMP3 apparatus (Stuart-Scientific, now Merck KGaA, Darmstadt, Germany) and are uncorrected. ^1^H-, ^13^C-, ^13^C DEPT-135, gs 2D HSQC and gs 2D HMBC NMR spectra were recorded in CDCl_3_ or MeOD solutions, and are referenced to the residual peaks of CHCl_3_ at δ = 7.26 ppm and δ = 77.00 ppm for ^1^H and ^13^C, respectively and CD_3_OD at δ = 3.30 ppm and δ = 49.00 ppm for ^1^H and ^13^C, respectively, on an Avance 400 Digital NMR spectrometer (Bruker, Rheinstetten, Germany) operating at 400.1 MHz for ^1^H and 100.6 MHz for ^13^C. Chemical shifts are reported in δ ppm and coupling constants (*J*) are given in Hz, multiplicities are reported as follows: singlet (s), doublet (d), doublet of doublets (dd), doublet of triplets (dt), triplet (t), quartet (q), multiplet (m). IR spectra were recorded as KBr disks in a FT-IR 6700 spectrometer (Nicolet, Thermo Scientific, San Jose, CA, USA) and frequencies are reported in cm^−1^. High-resolution mass spectra (HRMS-ESI) were recorded in a Exactive Plus mass spectrometer (Thermo Scientific, Waltham, MA, USA). The analysis for the reaction products was performed with the following relevant parameters: heater temperature, 50 °C; sheath gas flow, 5 (arbitrary unit); sweep gas flow rate, 0 (arbitrary unit) and spray voltage, 3.0 kV at negative mode. The accurate mass measurements were performed at a resolving power: 140,000 FWHM at range *m*/*z* 300–500. Optical rotations were measured on a Model AA-5 polarimeter (Optical Activity, Ltd., NJ, USA) with a sodium lamp using a l = 0.1 dm cell and are reported as follows: ${\left[\alpha \right]}_{D}^{{}^{\circ}C}$ (c (g/100 mL), solvent). For analytical TLC, silica gel 60 in 0.25 mm layer was used and TLC spots were detected by heating after spraying with 25% H_2_SO_4_ in H_2_O. Chromatographic separations were carried out by conventional column on silica gel 60 (230–400 mesh) using EtOAc-hexane gradients of increasing polarity. All organic extracts were dried over anhydrous magnesium sulfate and evaporated under reduced pressure, below 40 °C.

### 3.2. X-ray Crystal Structure Determination

A suitable single crystal of compound **22** was mounted on a MiTeGen MicroMount (MiTeGen, Lansing, NY, USA) in a random orientation. Diffraction data was collected at 120 K on a D8 VENTURE diffractometer (Bruker, Rheinstetten, Germany) equipped with a bidimensional CMOS Photon100 detector, using graphite monochromated Cu-Kα radiation (*λ* = 1.54178 Å). The diffraction frames were integrated using the APEX2 package. The structure of **22** was solved using Olex2 [51], with the olex2.solve structure solution program using Charge Flipping [52] and refined with full-matrix least-square methods based on *F*^2^ (*SHELXL*) [53]. Non-hydrogen atoms were refined with anisotropic displacement parameters. All hydrogen atoms were included in their calculated positions, assigned fixed isotropic thermal parameters and constrained to ride on their parent atoms. A summary of the details about crystal data, collection parameters and refinement are documented in Supplementary Material, and additional crystallographic details are in the CIF files. CCDC 1583718 contains the supplementary crystallographic data for this paper. These data can be obtained free of charge via http://www.ccdc.cam.ac.uk/conts/retrieving.html (or from the CCDC, 12 Union Road, Cambridge CB2 1EZ, UK; Fax: +44-1223-336033; E-mail: deposit@ccdc.cam.ac.uk. ORTEP view was drawn using OLEX2 software [51].

### 3.3. Synthesis

*3α-Acetoxy-24-nor-5α-cholan-22-en-6-one* (**20**). To a solution of **17** (4.00 g, 9.25 mmol) in dry benzene (150 mL) were added Cu(OAc)_2_ (0.29 g, 1.60 mmol) and pyridine (1.5 mL). Then, under reflux, Pb(OAc)_4_ (9.75 g, 22.0 mmol) was added in four portions at hourly intervals. After the addition was completed, the reaction was continued for 1 h. The end of reaction was verified by TLC, and then the mixture was filtered, and the solvent was evaporated under reduced pressure. The crude was re-dissolved in DCM (8 mL) and chromatographed on silica gel with PE/EtOAc mixtures of increasing polarity (19.8:0.2 → 15.8:4.2). Compound **20** (2.67 g, 75% yield) was obtained as a colorless solid: m.p. 64.0–66.1°C (hexane/Et_2_O = 1/1); ${\left[\alpha \right]}_{D}^{19}$ = −21.2° (c = 2.36, MeOH); ^1^H-NMR (CDCl_3_) δ 5.66 (ddd, *J* = 17.0; 10.2 and 8.4 Hz, 1H, H-22), 5.12 (m, 1H, H-3), 4.93 (dd, *J* = 17.0 and 1.8 Hz, 1H, H*_trans_*-23), 4.83 (dd, *J* = 10.2 and 1.8 Hz, 1H, H*_cis_*-23), 2.56 (dd, *J* = 12.1 and 3.2 Hz, 1H, H-5), 2.31 (dd, *J* = 13.1 and 4.5 Hz, 1H, H-7α), 2.04 (s, 3H, CH_3_CO), 1.04 (d, *J* = 6.6 Hz, 3H, H-21), 0.748 (s, 3H, H-19), 0.694 (s, 3H, H-18); ^13^C-NMR (CDCl_3_) see Table 1; IR ν_max_: 3082 (CH=CH_2_); 2946; 2909; 2868 and 2849 (C-H), 1740 (C=O), 1708 (C=O), 1637 (C=C), 1263 (C-O), 1021 (C-O), 988 (CH=CH_2_), 926 (CH=CH_2_) cm^−1^. HRMS-ESI (positive mode): *m*/*z* calculated for C_25_H_38_O_3_: 386.2821 [M]^+^; found 387.2874 [M + H]^+^.

*3α-Acetoxy-22(S), 23-dihydroxy-24-nor-5α-cholan-6-one* (**21a**) and *3α-acetoxy-22(R), 23-dihydroxy-24-nor-5α-cholan-6-one* (**21b**). To a solution of **20** (2.50 g, 6.47 mmol) in acetone (150 mL) was added NMO (0.45 g, 3.84 mmol). Then the mixture was homogenized by magnetic stirring and 2.0 mL of 4% OsO_4_ (0.210 mmol) was added dropwise with stirring for 36 h at room temperature. The end of the reaction was verified by TLC. Then the solvent was removed (up to 25 mL approximate volume) and water (25 mL) and Na_2_S_2_O_3_·5H_2_O (25 mL saturated solution) were added. The organic layer was extracted with EtOAc (2 × 30 mL), washed with water (2 × 50 mL), dried over Na_2_SO_4_, and filtered. The solvent was evaporated under reduced pressure. The crude was re-dissolved in DCM (10 mL) and chromatographed on silica gel with PE/EtOAc mixtures of increasing polarity (19.8:0.2 → 9.8:10.2). A mixture of **21a**/**21b** = 7.5/1.0 was obtained (1.97 g, 72% yield). Recrystallization of this mixture (MeOH/Et_2_O = 3/1) allows for compound **21a** to be obtained as colorless solid (1.74 g, 64% yield): m.p. 250.1–253.8 °C; ${\left[\alpha \right]}_{D}^{19}$ = −7.41° (c = 5.40, CHCl_3_); ^1^H-NMR (CDCl_3_) δ 5.11 (m, 1H, H-3), 3.79 (dt, *J* = 9.6 and 3.6 Hz, 1H, H-22), 3.64 (dd, *J* = 10.8 and 3.6 Hz, 1H, H-23a), 3.51 (dd, *J* = 10.8 and 9.6 Hz, 1H, H-23b), 2.55 (dd, *J* = 12.1 and 3.2 Hz, 1H, H-5), 2.31 (dd, *J* = 13.1 and 4.5 Hz, 1H, H-7α), 2.03 (s, 3H, CH_3_CO), 0.954 (d, *J* = 6.9 Hz, 3H, H-21), 0.735 (s, 3H, H-19), 0.675 (s, 3H, H-18); ^13^C-NMR (CDCl_3_) see Table 1; IR ν_max_: 3519 (O-H), 2941 and 2885 (C-H), 1732 (C=O), 1708 (C=O), 1278 (C-O), 1050 (C-O) cm^−1^; HRMS-ESI (negative mode): *m*/*z* calculated for C_25_H_40_O_5_: 420.2876 [M]^+^; found 419.2811 [M − H]^−^.

*3α-Acetoxy-22(S)-hydroxy-24-nor-5α-cholan-6-oxo-23-benzoate* (**22**). Compound **21a** (0.5 g, 1.19 mmol) was dissolved in DCM (25 mL) and pyridine (1.0 mL). Later DMAP (5.0 mg) and PhCOCl (0.5 mL, 4.30 mmol) were added with slow stirring at room temperature. The end of the reaction was verified by TLC (2 h), solvent volume was reduced to about 10 mL, and then EtOAc (20 mL) were added. The organic layer was washed with 5% KHSO_4_ (2 × 5 mL) and water (2 × 10 mL), dried over Na_2_SO_4_ and filtered. The solvent was evaporated under reduced pressure. The crude was redissolved in DCM (5 mL) and chromatographed on silica gel with PE/EtOAc mixtures of increasing polarity (19.8:0.2 → 14.2:5.8). Compound **22** (0.59 g, 93.8% yield) was obtained as a colorless solid: m.p. 210.5–211.7 °C (MeOH/Et_2_O = 1/2); ${\left[\alpha \right]}_{D}^{19}$ = −17.1° (c = 1.75, CHCl_3_); ^1^H-NMR (CDCl_3_) δ 8.05 (d, *J* = 7.4 Hz, 2H, H*_Ar_*-2’), 7.58 (t, *J* = 7.4 Hz, 1H, H*_Ar_*-4’), 7.46 (t, *J* = 7.4 Hz, 2H, H*_Ar_*-3’), 5.12 (m, 1H, H-3), 4.50 (dd, *J* = 11.4 and 1.7 Hz, 1H, H-23a), 4.20 (dd, *J* = 11.3 and 4.2 Hz, 1H, H-23b), 4.06 (m, 1H, H-22), 2.57 (dd, *J* = 12.0 and 2.9 Hz, 1H, H-5), 2.32 (dd, *J* = 13.1 and 4.5 Hz, 1H, H-7α), 2.04 (s, 3H, CH_3_CO), 1.06 (d, *J* = 6.9 Hz, 3H, H-21), 0.748 (s, 3H, H-19), 0.709 (s, 3H, H-18); ^13^C-NMR (CDCl_3_) (see Table 1); IR ν_max_: 3502 (O-H), 2951, 2936, 2893 and 2870 (C-H), 1730 (C=O), 1715 (C=O), 1693 (C=O), 1601 (C=C Ar), 1276 (C-O), 1070 (C-O), 716 (C-H Ar) cm^−1^; HRMS-ESI (positive mode): *m*/*z* calculated for C_32_H_44_O_6_: 524.3138 [M]^+^; found 525.3186 [M + H]^+^. 

*3α-22(S), 23-Trihydroxy-24-nor-5α-cholan-6-one* (**9**). To a solution of **21a** (0.5 g, 1.19 mmol) in MeOH (30 mL) was added K_2_CO_3_ (0.493 g, 3.57 mmol), then the suspension was stirred at room temperature for 3 h. The end of the reaction was verified by TLC. Then the solvent was removed to dryness and the residue acidified with 2% HCl (20 mL). The obtained solid was filtered and washed with 5% NaHCO_3_ (20 mL) and water (2 × 10 mL) and dried. Compound **9** (0.437 g, 97% yield) was obtained as a colorless solid: m.p. 227.0–229.1 °C (MeOH/Et_2_O = 3/1); ${\left[\alpha \right]}_{D}^{19}$ = −3.67° (c = 2.73, MeOH); ^1^H-NMR (CD_3_OD) δ 4.04–4.03 (m, 1H, H-3), 3.71 (dt, *J* = 8.9 and 3.2 Hz, 1H, H-22), 3.60 (dd, *J* = 11.3 and 2.7 Hz, 1H, H-23a), 3.40 (dd, *J* = 11.3 and 8.9 Hz, 1H, H-23b), 2.74 (t, *J* = 7.9 Hz, 1H, H-5), 2.21 (dd, *J* = 13.1 and 4.8 Hz, 1H, H-7α), 2.11 (t, *J* = 13.1 Hz, 1H, H-7α), 2.04 (dt, *J* = 12.2 and 2.2 Hz, 1H, H-12α), 0.943 (d, *J* = 6.9 Hz, 3H, H-21), 0.732 (s, 3H, H-19), 0.716 (s, 3H, H-18); ^13^C-NMR (CD_3_OD) see Table 1; IR ν_max_: 3387 (O-H), 2940, 2906 and 2871 (C-H), 1700 (C=O), 1246 (C-O), 1050 (C-O), 754 (CH) cm^−1^; HRMS-ESI (positive mode): *m*/*z* calculated for C_23_H_38_O_4_: 378.2770 [M]^+^; found 379.2823 [M + H]^+^.

*3α-22(S), 23-Triacetoxy-24-nor-5α-cholan-6-one* (**23**). Compound **21a** (2.0 g, 4.76 mmol) was dissolved in DCM (30 mL) and pyridine (3.0 mL). Later DMAP (5.0 mg) and Ac_2_O (1 mL, 10.6 mmol) were added to the solution and the reaction mixture was stirred at room temperature. The end of the reaction was verified by TLC (30 min), volume of solvent was reduced to about 5 mL and extracted with EtOAc (2 × 10 mL). The organic layer was washed with 5% KHSO_4_ (2 × 5 mL) and water (2 × 10 mL), dried over Na_2_SO_4_ and filtered. The solvent was evaporated under reduced pressure. The crude was redissolved in DCM (5 mL) and chromatographed on silica gel with PE/Et_2_O mixtures of increasing polarity (19.8:0.2 → 11.8:8.2). Compound **23** (2.33 g, 97% yield) was obtained as a colorless solid: m.p. 136.1–137.6 °C (Et_2_O/hexane); ${\left[\alpha \right]}_{D}^{19}$ = +2.53° (c = 3.95, CHCl_3_); ^1^H-NMR (CDCl_3_) δ 5.10–5.09 (m, 2H, H-3 and H-22), 4.31 (dd, *J* = 11.9 and 1.9 Hz, 1H, H-23a), 3.99 (dd, *J* = 11.9 and 9.4 Hz, 1H, H-23b), 2.54 (dd, *J* = 12.1 and 3.1 Hz, 1H, H-5), 2.30 (dd, *J* = 13.1 and 4.4 Hz, 1H, H-7α), 2.06 (s, 3H, AcO), 2.04 (s, 3H, AcO), 2.03 (s, 3H, AcO), 0.973 (d, *J* = 7.0 Hz, 3H, H-21), 0.723 (s, 3H, H-19), 0.651 (s, 3H, H-18); ^13^C-NMR (CDCl_3_) (see Table 1); IR ν_max_: 2945, 2908 and 2871 (C-H), 1737 (C=O), 1711 (C=O), 1369 (CH_3_), 1242 (C-O), 1224 (C-O), 1051 (C-O), 757 (C-H) cm^−1^; HRMS-ESI (positive mode): *m*/*z* calculated for C_29_H_44_O_7_: 504.3087 [M]^+^; found 505.3134 [M + H]^+^.

*3α-22(S), 23-Triacetoxy-24-nor-B-homo-7-oxa-5α-cholan-6-one* (**24**) and 3α-22(*S*), 23-triacetoxy-24-nor-*B*-homo-6-oxa-5α-cholan-6-one (**25**). Preparation of oxidant: 1.0 mL of H_2_O_2_ (30%), (9.77 mmol) was slowly dripped into a solution of (CF_3_CO)_2_O (1.20 mL, 8.52 mmol) at 0 °C, diluted with CHCl_3_ (3 mL) and stirred for 30 min. The oxidant mixture (3.0 mL) was slowly added to the solution of compound 23 (1.00 g, 1.98 mmol in 10 mL of CHCl_3_) at 0 °C and slowly stirred in N_2_ atmosphere for 24 h. The end of reaction was verified by TLC, the mixture was filtered, then concentrated in a rotary evaporator to a volume of approximately 10 mL. Then Et_2_O (40 mL) was added and the organic layer was washed with saturated NaHCO_3_ solution (2 × 20 mL), water (2 × 15 mL), then dried over Na_2_SO_4_, and filtered. The solvent was evaporated and the crude was re-dissolved in DCM (5 mL) and chromatographed on silica gel with hexane/Et_2_O mixtures of increasing polarity (0.2:50.0 → 23.8:26.2). Three fractions were obtained. Fraction I (less polar): 0.363 g (35% yield), compound **24**. Fraction II (medium polarity): 0.285 g, mixture of compounds **24** and **25**. Fraction III (more polar): 0.146 g (14% yield) compound **25**. 

Compound **24** was obtained as a colorless solid: m.p. 90.3–91.8 °C (Hexane/Et_2_O = 2/1); ${\left[\alpha \right]}_{D}^{19}$ = +50.9° (c = 4.13, CHCl_3_); ^1^H-NMR (CDCl_3_) δ 5.11–5.09 (m, 2H, H-3 and H-22), 4.32 (dd, *J* = 11.9 and 1.9 Hz, 1H, H-23a), 4.13–4.04 (m, 2H, H-7α and H-7α), 3.99 (dd, *J* = 11.9 and 9.4 Hz, 1H, H-23b), 3.03 (dd, *J* = 12.2 and 4.3 Hz, 1H, H-5), 2.08 (ddd, *J* = 16.2, 12.2 and 2.70 Hz, 1H, H-4α), 2.08 (s, 6H, AcO), 2.05 (s, 3H, AcO), 0.979 (d, *J* = 6.8 Hz, 3H, H-21), 0.897 (s, 3H, H-19), 0.701 (s, 3H, H-18); ^13^C-NMR (CDCl_3_) (see Table 2); IR ν_max_: 2965, 2946, 2909 and 2872 (C-H), 1735 (C=O), 1438 (C-H), 1368 (CH_3_), 1242 (C-O), 1182 (C-O), 1052 (C-O), 754 (C-H) cm^−1^; HRMS-ESI (positive mode): *m*/*z* calculated for C_29_H_44_O_8_: 520.3036 [M]^+^; found 521.3085 [M + H]^+^.

Compound **25** was obtained as a colorless solid: m.p. 169.9–170.8 °C (Hexane/Et_2_O = 2/1); ${\left[\alpha \right]}_{D}^{25}$ = +61.4° (c = 0.44, MeOH); ^1^H-NMR (CDCl_3_) δ 5.14–5.09 (m, 2H, H-3 and H-22), 4.46 (dd, *J* = 11.3 and 5.3 Hz, 1H, H-5), 4.32 (dd, *J* = 12.0 and 2.0 Hz, 1H, H-23a), 3.99 (dd, *J* = 12.0 and 9.4 Hz, 1H, H-23b), 2.55–2.43 (m, 2H, H-7α and H-7α), 2.07 (s, 6H, AcO), 2.05 (s, 3H, AcO), 0.971 (d, *J* = 6.9 Hz, 3H, H-21), 0.901 (s, 3H, H-19), 0.695 (s, 3H, H-18); ^13^C-NMR (CDCl_3_) see Table 2; IR ν_max_: 2965, 2949 and 2871 (C-H), 1743 (C=O), 1736 (C=O), 1725 (C=O), 1445 (C-H), 1368 (CH_3_), 1258 (C-O), 1239 (C-O), 1225 (C-O), 1044 (C-O), 1022 (C-O), 754 (C-H) cm^−1^; HRMS-ESI (positive mode): *m*/*z* calculated for C_29_H_44_O_8_: 520.3036 [M]^+^; found 521.3082 [M + H]^+^.

*3α-22(S), 23-Trihydroxy-24-nor-B-homo-7-oxa-5α-cholan-6-one* (**18**). To a solution of **24** (0.15 g, 0.288 mmol) in MeOH (20 mL) was added K_2_CO_3_ (0.050 g, 0.307 mmol), then the suspension was stirred at room temperature for 3 h. The end of the reaction was verified by TLC. Then the solvent was removed to dryness and the residue acidified with 2% HCl (10 mL). The obtained solid was filtered and washed with 5% NaHCO_3_ (20 mL) and water (2 × 10 mL) and dried. Compound **18** (0.098 g, 86% yield) was obtained as a colorless solid: m.p. 91.8–94.5 °C (MeOH/Et_2_O = 3/1); ${\left[\alpha \right]}_{D}^{19}$ = +54.2° (c = 2.95, MeOH); ^1^H-NMR (CDCl_3_) δ 4.19–4.15 (m, H-3), 4.08–4.07 (m, 2H, H-7α and H-7α), 3.79 (dt, *J* = 9.3 and 3.1 Hz, 1H, H-22), 3.64 (dd, *J* = 11.0 and 2.8 Hz, 1H, H-23a), 3.52 (dd, *J* = 11.0 and 9.4 Hz, 1H, H-23b), 3.17 (dd, *J* = 12.3 and 4.4 Hz, 1H, H-5), 2.13 (ddd, *J* = 13.7, 12.4 and 2.7 Hz, 1H, H-4α), 1.98 (dt, *J* = 12.6 and 3.3 Hz, 1H, H-12α), 0.950 (d, *J* = 6.9 Hz, 3H, H-21), 0.888 (s, 3H, H-19), 0.710 (s, 3H, H-18); ^13^C-NMR (CDCl_3_/CD_3_OD = 2/1) see Table 2; IR ν_max_: 3400 (O-H); 2959; 2941; 2902 and 2870 (C-H); 1709 (C=O); 1315 (C-H); 1249 (C-O); 1183 (C-O), 1065 (C-O); 1048 (C-O), 753 (C-H) cm^−1^; HRMS-ESI (positive mode): *m*/*z* calculated for C_23_H_38_O_5_: 394.2719 [M]^+^; found 395.2771 [M + H]^+^.

*3α-22(S), 23-Trihydroxy-24-nor-B-homo-6-oxa-5α-cholan-6-one* (**19**). Compound **19** was obtained from **25** by the same method described above. Compound **25** (0.15 g, 0.288 mmol), MeOH (20 mL), K_2_CO_3_ (0.050 g, 0.307 mmol). Compound **25** (0.092 g, 81% yield), colorless solid: m.p. 227.4–229.5 °C (MeOH/Et_2_O =3/1); ${\left[\alpha \right]}_{D}^{19}$ = +27.1° (c = 1.48, MeOH); ^1^H-NMR (CDCl_3_) δ 4.60 (dd, *J* = 11.3 and 5.3 Hz, 1H, H-5), 4.23–4.21 (m, 1H, H-3), 3.79 (dt, *J* = 9.4 and 3.1 Hz, 1H, H-22), 3.64 (dd, *J* = 10.9 and 2.6 Hz, 1H, H-23a), 3.50 (dd, *J* = 10.9 and 9.4 Hz, 1H, H-23b), 2.53–2.43 (m, 2H, H-7α and H-7α), 0.944 (d, *J* = 6.9 Hz, 3H, H-21), 0.892 (s, 3H, H-19), 0.701 (s, 3H, H-18); ^13^C-NMR (CDCl_3_/CD_3_OD = 2/1) see Table 2; IR ν_max_: 3386 (O-H); 2942; 2889; 2869 and 2851 (C-H); 1710 (C=O); 1278 (C-O); 1038 (C-O); 751 (C-H) cm^−1^; HRMS-ESI (negative mode): *m*/*z* calculated for C_23_H_38_O_5_: 394.2719 [M]^+^; found 393.2652 [M − H]^−^.

## 4. Conclusions

A new synthetic route has been used to obtain the known brassinosteroid analog **9** and new compounds **18**, **19**, **21a**, **22**–**25**. Compound **9** was obtained from **17** in a total yield of 46%, whereas new lactones analogues **18** and **19** were obtained from glycol **21a** in 29% and 11% total yields. Additionally, using 1D, 2D NMR, and HRMS we have achieved full structural determination of all compounds shown in Scheme 1 and Scheme 2. The absolute stereochemistry at position C-22 was established a (*S*) by X-ray crystallography studies of the benzoylated derivative **22**. This conclusion is in line with literature data reported for similar steroidal structures [14]. Finally, in order to establish a relationship between the side chain structure of BRs analogs and the promoting plant growth activity, additional changes on the side chain should be introduced.

## Figures and Tables

**Table 1:** δ(ppm) ^13^C-NMR (CDCl_3_, 100.6 MHz) for compounds **20**, **21a**, **22**, and **9**.

C	20	21a	22	9 *
1	32.38	32.17	32.36	32.88
2	28.19	27.20	27.41	28.73
3	68.85	68.70	68.81	66.02
4	25.27	25.05	25.26	28.53
5	52.58	52.38	52.58	52.84
6	211.81	211.72	211.62	214.49
7	46.75	46.49	46.69	47.61
8	37.92	37.74	37.90	39.45
9	53.79	52.68	52.90	54.19
10	41.28	41.07	41.23	42.62
11	21.06	20.87	21.06	22.19
12	39.36	39.19	39.41	40.76
13	42.94	43.16	43.41	44.46
14	55.41	53.51	53.72	55.08
15	25.01	24.78	25.00	28.45
16	23.91	23.85	24.02	25.07
17	56.80	56.15	56.41	57.56
18	12.18	11.56	11.81	12.14
19	12.41	12.20	12.40	12.67
20	41.10	39.87	40.33	42.01
21	20.04	12.86	12.90	13.42
22	144.89	73.69	71.77	75.19
23	111.84	62.22	66.39	63.19
CH_3_CO	170.26	170.15	170.27	-
CH_3_CO	21.41	21.21	21.41	-
CH_3_CO	-	-	-	-
CH_3_CO	-	-	-	-
CH_3_CO	-	-	-	-
CH_3_CO	-	-	-	-
COAr	-	-	167.01	-
1’	-	-	129.86	-
2’	-	-	129.63	-
3’	-	-	128.45	-
4’	-	-	133.22	-

* The ^13^C-NMR spectrum of compound **9** was recorded in MeOD solution.

**Table 2:** δ(ppm) ^13^C-NMR (CDCl_3_, 100.6 MHz) for compounds **18**, **19**, **23**–**25**.

C	23	24	25	18 *	19 *
1	32.35	33.66	31.84	32.78	31.06
2	27.01	26.95	26.65	24.84	25.25
3	68.79	68.38	69.56	64.32	65.60
4	25.24	29.74	32.95	32.50	35.39
5	52.55	42.57	79.68	41.67	80.04
6	211.49	175.97	174.64	177.44	176.08
7	46.63	70.31	38.12	70.43	37.88
8	37.85	39.42	38.31	39.54	39.89
9	52.84	58.31	57.97	58.15	57.77
10	41.18	36.14	39.55	36.17	39.64
11	21.03	22.10	22.17	22.03	22.02
12	39.38	39.53	39.69	40.03	39.48
13	43.39	43.03	43.09	42.91	42.91
14	53.67	52.80	55.13	52.70	54.94
15	24.98	25.13	25.33	27.87	27.46
16	23.94	24.83	24.83	27.20	26.76
17	56.37	51.10	53.14	50.98	52.97
18	11.81	11.57	11.59	11.41	11.32
19	12.38	14.54	11.59	14.39	11.32
20	38.32	38.32	34.78	39.33	34.77
21	13.37	13.28	13.29	12.77	12.66
22	73.97	73.80	73.80	73.57	73.50
23	62.39	62.30	62.35	62.08	62.02
CH_3_CO	171.09	171.07	171.12	-	-
CH_3_CO	21.39	21.36	21.32	-	-
CH_3_CO	170.42	170.40	170.41	-	-
CH_3_CO	21.23	21.21	21.24	-	-
CH_3_CO	170.25	170.28	170.17	-	-
CH_3_CO	20.86	20.85	20.88	-	-

* The ^13^C-NMR spectrum of compound **18** and **19** were recorded in CDCl_3_/MeOD 2/1, solution.

## Supplementary Materials

The following are available online, X-ray structure of compound **22** (CIF); Spectra ^1^H and ^13^C-NMR of compounds **9**, **18**–**20**, **21a**, **23**–**25** (PDF); Spectra HRMS of compounds **9**, **18**–**20**, **21a**, **23**–**25** (PDF).
