# Supplementary material for: Synthesis of 2-Deoxybrassinosteroids Analogs with 24-nor, 22(S)-23-Dihydroxy-Type Side Chains from Hyodeoxycholic Acid
Source: Molecules. 2018 May 29;23(6):1306. doi: 10.3390/molecules23061306 (PMC6099934; doi:10.3390/molecules23061306)
Supplement: Supplementary file 1 [file molecules-23-01306-s001.pdf]

Synthesis of 2-deoxybrassinosteroids analogs with 24-nor, 22(*S*)-23-dihydroxy-type side chain, from hyodeoxycholic acid

Rodrigo Carvajal[1], Cesar González[1], Andrés F. Olea[2,*], Mauricio Fuentealba[3] and Luis Espinoza[1,*]

[1] Departamento de Química, Universidad Técnica Federico Santa María, Avenida España 1680, Valparaíso 2340000, Chile; rodrigo.carvajal@postgrado.usm.cl; cesar.gonzalez@usm.cl; luis.espinozac@usm.cl

[2] Instituto de Ciencias Químicas Aplicadas, Facultad de Ingeniería, Universidad Autónoma de Chile, Santiago 8910339, Chile; andres.olea@uautonoma.cl

[3] Laboratorio de Cristalografía, Pontificia Universidad Católica de Valparaíso, Avenida Universidad 330, Curauma, Valparaíso, Chile

1. X-Ray data, bond distances, and angles for **22** S1

2. Copies of ^{1}H and ^{13}C NMR Spectra S2

3. Copies of HRMS S3

S1. X-Ray data, bond distances, and angles for **22**

Table S1. Crystal data and structure refinement for compound 22

Identification code	cu_MF_RE1_0m
Empirical formula	$C_{32}H_{44}O_6$
Formula weight	524.67
Temperature/K	120.0
Crystal system	orthorhombic
Space group	$P2_12_12_1$
a/Å	6.3224(3)
b/Å	11.7333(5)
c/Å	38.4435(17)
α/°	90
β/°	90
γ/°	90
Volume/Å^3	2851.8(2)
Z	4
ρ_{calc}g/cm^3	1.222
μ/mm^{-1}	0.663
F(000)	1136.0
Crystal size/mm^3	0.253 × 0.132 × 0.048
Radiation	CuKα (λ = 1.54178)
2Θ range for data collection/°	4.596 to 149.602
Index ranges	-7 ≤ h ≤ 7, -13 ≤ k ≤ 14, -47 ≤ l ≤ 45
Reflections collected	22330
Independent reflections	5751 [R_{int} = 0.1434, R_{sigma} = 0.0918]
Data/restraints/parameters	5751/0/348
Goodness-of-fit on F^2	1.101
Final R indexes [I>=2σ (I)]	R_1 = 0.0703, wR_2 = 0.1348
Final R indexes [all data]	R_1 = 0.1104, wR_2 = 0.1538
Largest diff. peak/hole / e Å^{-3}	0.42/-0.23
Flack parameter	-0.2(3)

Table S2. Bond lengths for compound 22

Atom	Atom	Length/Å	Atom	Atom	Length/Å
O5	C31	1.356(6)	C20	C21	1.527(6)
O5	C3	1.476(7)	C20	C22	1.552(7)
O4	C24	1.202(6)	C1	C10	1.546(7)
O3	C24	1.338(6)	C1	C2	1.514(7)
O3	C23	1.446(6)	C31	C32	1.475(8)
O6	C31	1.209(7)	C15	C16	1.543(6)
C17	C13	1.562(7)	C10	C5	1.562(7)
C17	C20	1.535(6)	C10	C19	1.544(7)
C17	C16	1.550(6)	C23	C22	1.522(7)
C11	C9	1.533(7)	C5	C6	1.523(7)
C11	C12	1.538(7)	C5	C4	1.522(7)
C13	C12	1.530(6)	C6	O1	1.221(6)
C13	C14	1.545(6)	C4	C3	1.515(7)
C13	C18	1.532(6)	C2	C3	1.518(7)
C9	C10	1.543(7)	C26	C25	1.392(7)
C9	C8	1.542(6)	C26	C27	1.394(8)
C14	C15	1.533(6)	C22	O2	1.446(6)
C14	C8	1.514(7)	C25	C30	1.396(7)
C7	C6	1.500(7)	C30	C29	1.391(8)
C7	C8	1.543(6)	C29	C28	1.370(9)
C24	C25	1.499(7)	C28	C27	1.376(9)

Table S3. Bond angles for compound 22

Atom	Atom	Atom	Angle/°	Atom	Atom	Atom	Angle/°
C31	O5	C3	118.3(4)	C9	C10	C1	109.9(4)
C24	O3	C23	116.1(4)	C9	C10	C5	107.3(4)
C20	C17	C13	119.3(4)	C9	C10	C19	111.4(4)
C20	C17	C16	112.9(4)	C1	C10	C5	107.0(4)
C16	C17	C13	103.4(4)	C19	C10	C1	110.4(4)
C9	C11	C12	112.9(4)	C19	C10	C5	110.7(4)
C12	C13	C17	117.3(4)	O3	C23	C22	112.2(4)
C12	C13	C14	106.6(4)	C6	C5	C10	111.3(4)
C12	C13	C18	110.0(4)	C4	C5	C10	113.7(4)
C14	C13	C17	98.4(4)	C4	C5	C6	113.6(4)
C18	C13	C17	110.4(4)	C7	C6	C5	115.3(4)
C18	C13	C14	113.7(4)	O1	C6	C7	122.1(5)
C11	C9	C10	115.2(4)	O1	C6	C5	122.5(5)
C11	C9	C8	111.3(4)	C3	C4	C5	111.3(4)

C8	C9	C10	111.3(4)	C1	C2	C3	111.9(5)
C13	C12	C11	111.5(4)	C25	C26	C27	119.5(6)
C15	C14	C13	104.6(4)	C23	C22	C20	112.2(4)
C8	C14	C13	115.3(4)	O2	C22	C20	108.1(4)
C8	C14	C15	118.4(4)	O2	C22	C23	108.1(4)
C6	C7	C8	114.7(4)	C9	C8	C7	110.3(4)
O4	C24	O3	125.2(5)	C14	C8	C9	109.1(4)
O4	C24	C25	123.9(5)	C14	C8	C7	109.8(4)
O3	C24	C25	111.0(5)	O5	C3	C4	109.4(4)
C17	C20	C22	112.4(4)	O5	C3	C2	105.7(4)
C21	C20	C17	111.5(4)	C4	C3	C2	112.4(5)
C21	C20	C22	111.1(4)	C26	C25	C24	122.4(5)
C2	C1	C10	113.8(4)	C26	C25	C30	119.5(5)
O5	C31	C32	111.4(5)	C30	C25	C24	118.0(5)
O6	C31	O5	122.4(6)	C29	C30	C25	120.1(6)
O6	C31	C32	126.2(5)	C28	C29	C30	119.9(6)
C14	C15	C16	103.9(4)	C29	C28	C27	120.7(6)
C15	C16	C17	106.4(4)	C28	C27	C26	120.3(6)

S2. Copies of ^{1}H and ^{13}C NMR Spectra

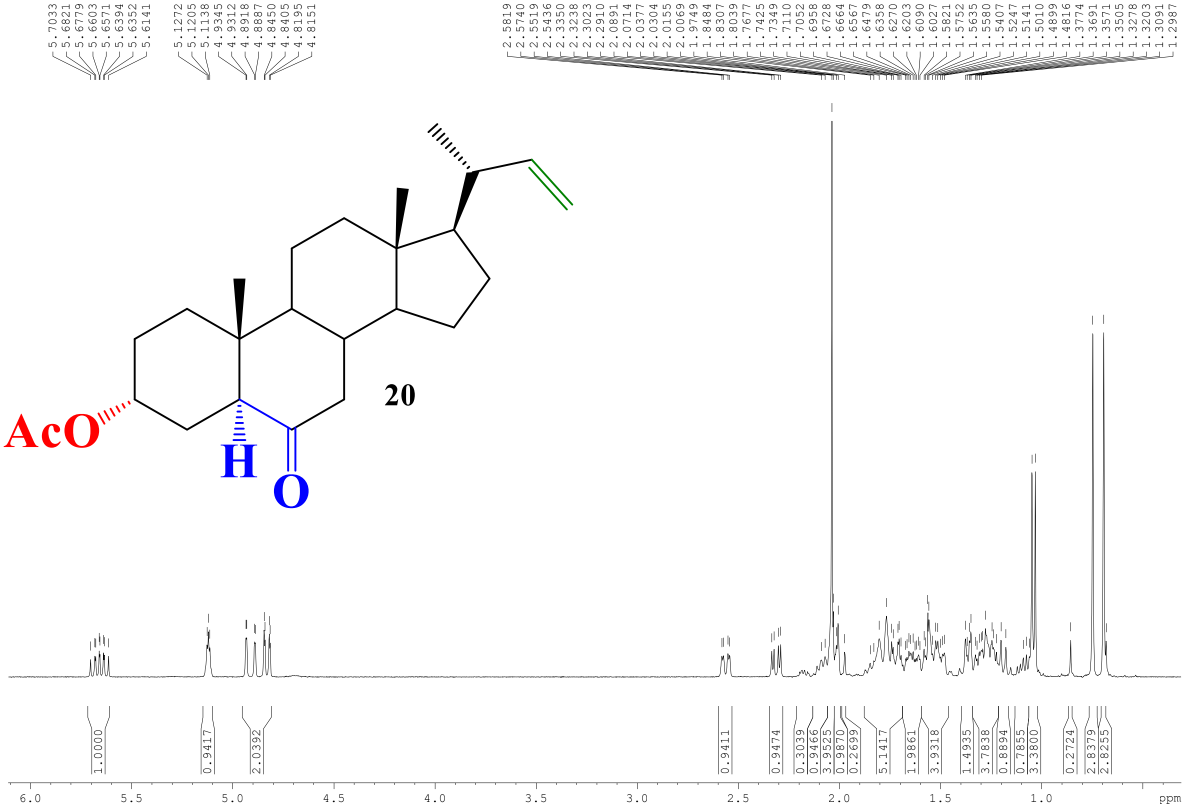

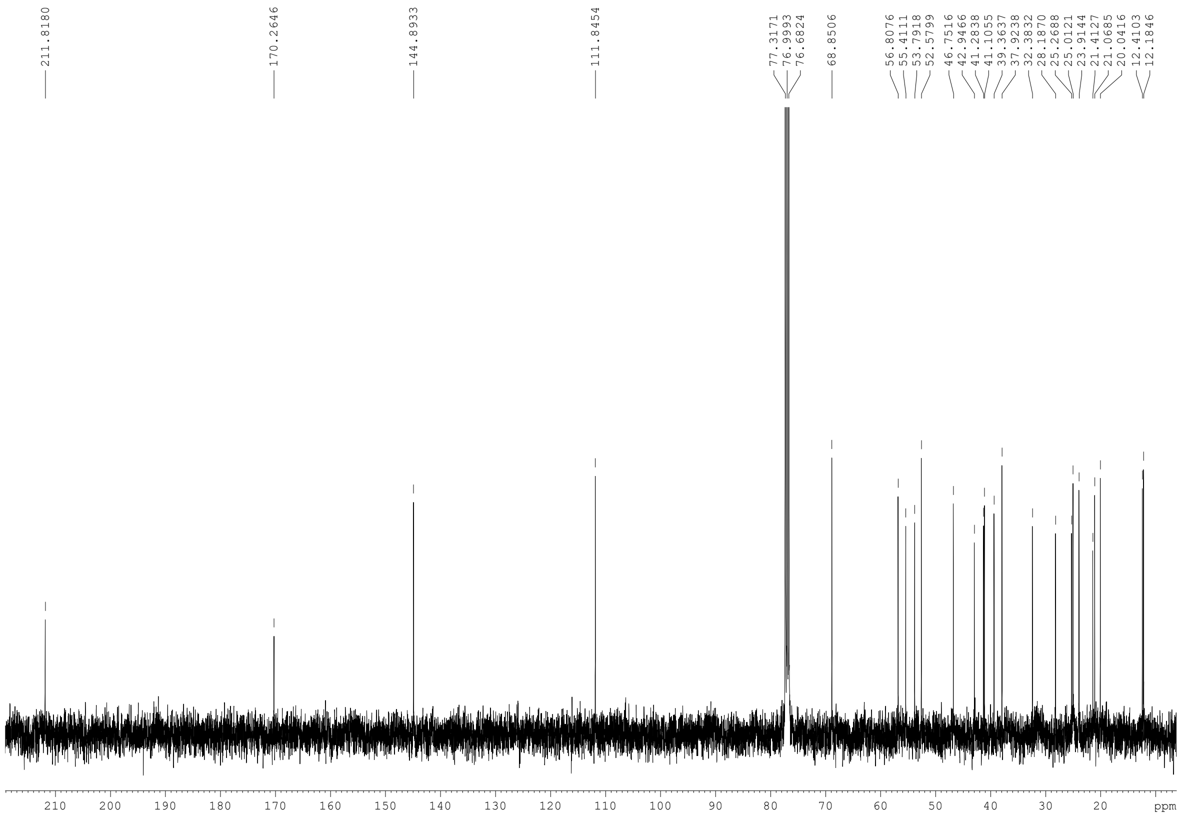

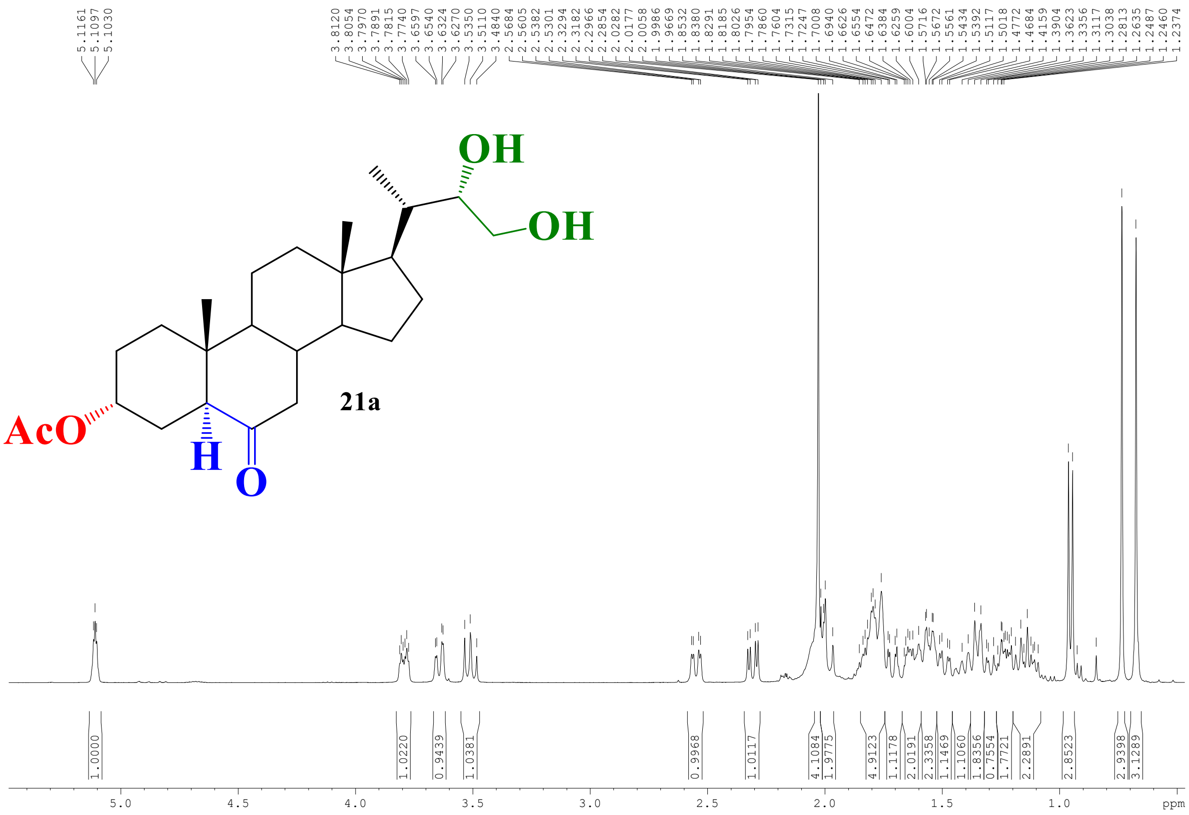
5.1161
5.1097
5.1030
3.8120
3.8054
3.7970
3.7891
3.7815
3.7740
3.6597
3.6540
3.6324
3.6270
3.5350
3.5110
3.4840
2.5684
2.5605
2.5382
2.5301
2.3294
2.3182
2.2966
2.2854
2.0282
2.0177
2.0058
1.9986
1.9669
1.8532
1.8380
1.8291
1.8185
1.8026
1.7954
1.7860
1.7604
1.7315
1.7247
1.7008
1.6940
1.6626
1.6554
1.6472
1.6384
1.6259
1.6004
1.5716
1.5672
1.5561
1.5434
1.5392
1.5117
1.5018
1.4772
1.4684
1.4159
1.3904
1.3623
1.3356
1.3117
1.3038
1.2813
1.2635
1.2487
1.2460
1.2374
OH
OH
AcO
H
O
21a
1.0000
1.0220
0.9439
1.0381
0.9968
1.0117
4.1084
1.9775
4.9123
1.1178
2.0191
2.3358
1.1469
1.1060
1.8356
0.7554
1.7721
2.2891
2.8523
2.9398
3.1289
5.0
4.5
4.0
3.5
3.0
2.5
2.0
1.5
1.0
ppm

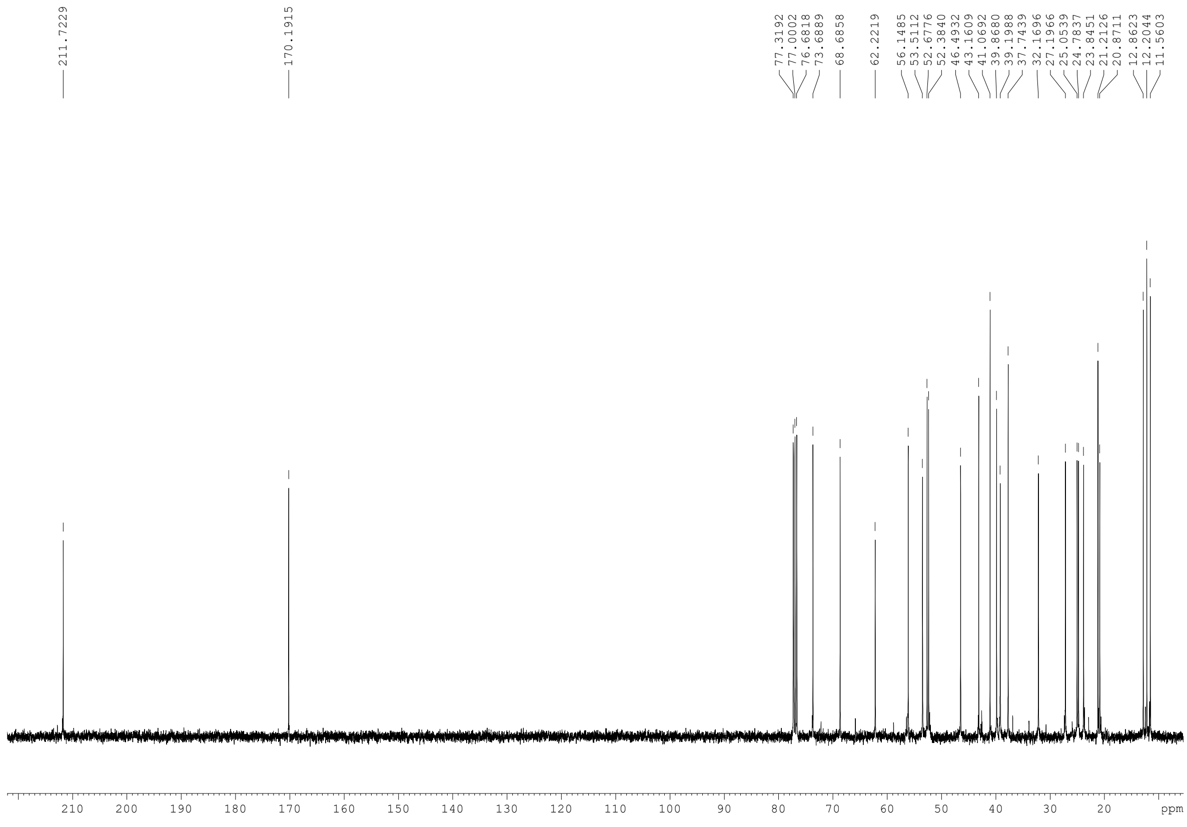
211.7229
170.1915
77.3192
77.0002
76.6818
73.6889
68.6858
62.2219
56.1485
53.5112
52.6776
52.3840
46.4932
43.1609
41.0692
39.8680
39.1988
37.7439
32.1696
27.1966
25.0539
24.7837
23.8451
21.2126
20.8711
12.8623
12.2044
11.5603
210
200
190
180
170
160
150
140
130
120
110
100
90
80
70
60
50
40
30
20
ppm

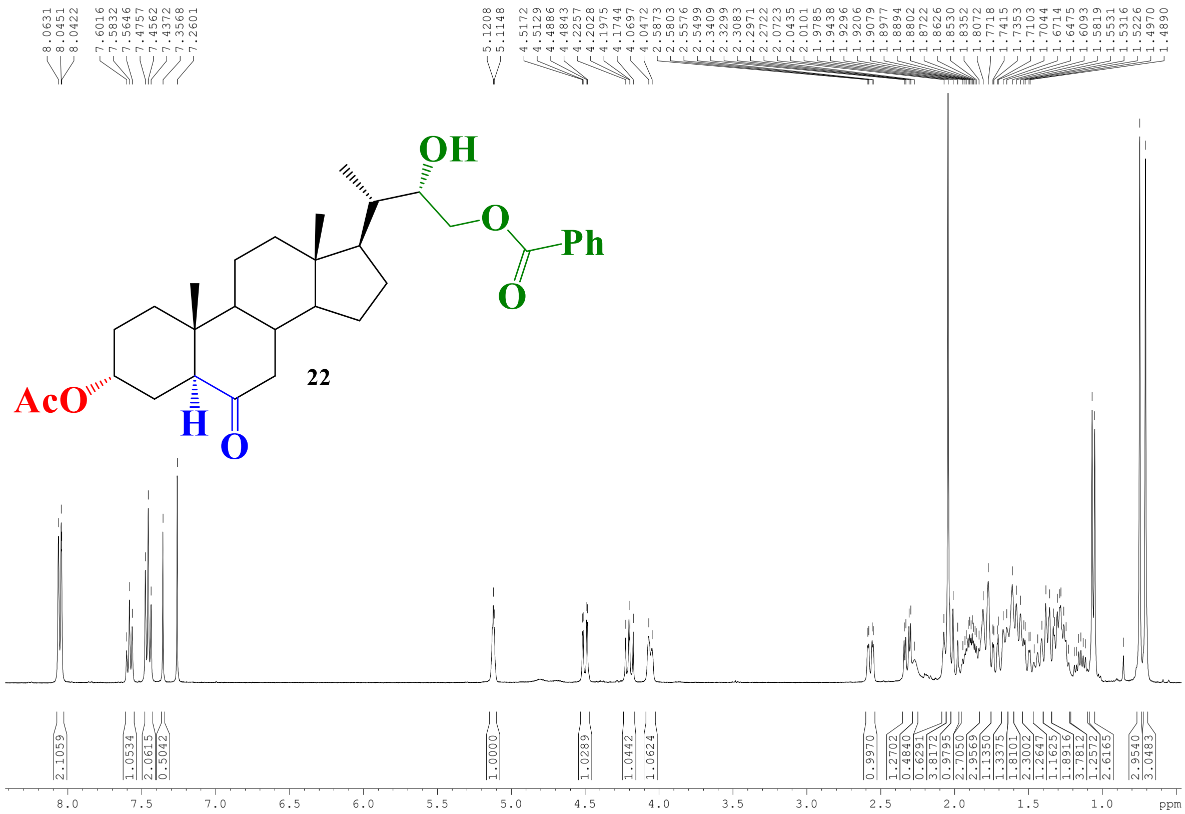
8.0631
8.0451
8.0422
7.6016
7.5832
7.5646
7.4757
7.4562
7.4372
7.3568
7.2601
5.1208
5.1148
4.5172
4.5129
4.4886
4.4843
4.2257
4.2028
4.1975
4.1744
4.0697
4.0472
2.5873
2.5803
2.5576
2.5499
2.3409
2.3299
2.3083
2.2971
2.2722
2.0723
2.0435
2.0101
1.9785
1.9438
1.9296
1.9206
1.9079
1.8977
1.8894
1.8802
1.8722
1.8626
1.8530
1.8352
1.8072
1.7718
1.7415
1.7353
1.7103
1.7044
1.6714
1.6475
1.6093
1.5819
1.5531
1.5316
1.5226
1.4970
1.4890
OH
O
Ph
O
AcO
H
O
22
2.1059
1.0534
2.0615
0.5042
1.0000
1.0289
1.0442
1.0624
0.9970
1.2702
0.4840
0.6291
3.8172
0.9795
2.7050
2.9569
1.1350
1.3375
1.8101
2.3002
1.2647
1.1625
1.8916
3.7812
1.2572
2.6165
2.9540
3.0483
8.0 7.5 7.0 6.5 6.0 5.5 5.0 4.5 4.0 3.5 3.0 2.5 2.0 1.5 1.0 ppm

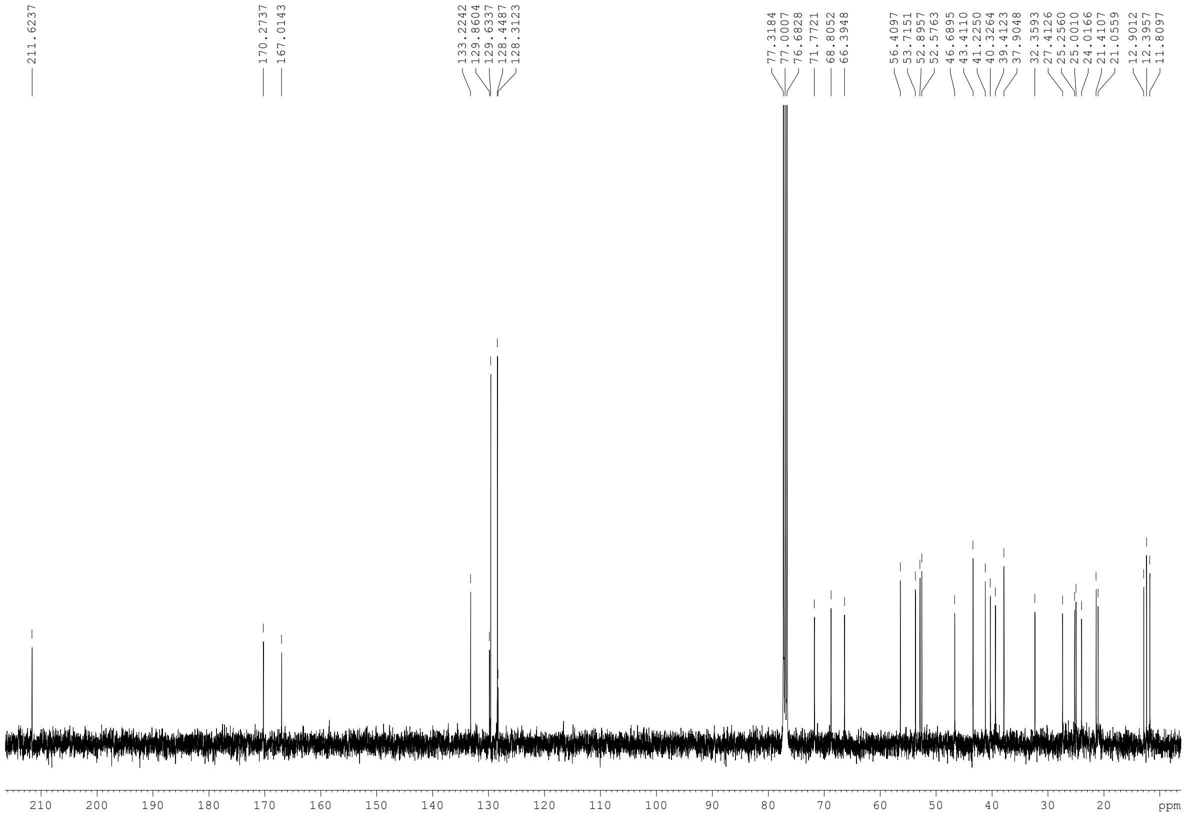
211.6237
170.2737
167.0143
133.2242
129.8604
129.6337
128.4487
128.3123
77.3184
77.0007
76.6828
71.7721
68.8052
66.3948
56.4097
53.7151
52.8957
52.5763
46.6895
43.4110
41.2250
40.3264
39.4123
37.9048
32.3593
27.4126
25.2560
25.0010
24.0166
21.4107
21.0559
12.9012
12.3957
11.8097
210 200 190 180 170 160 150 140 130 120 110 100 90 80 70 60 50 40 30 20 ppm

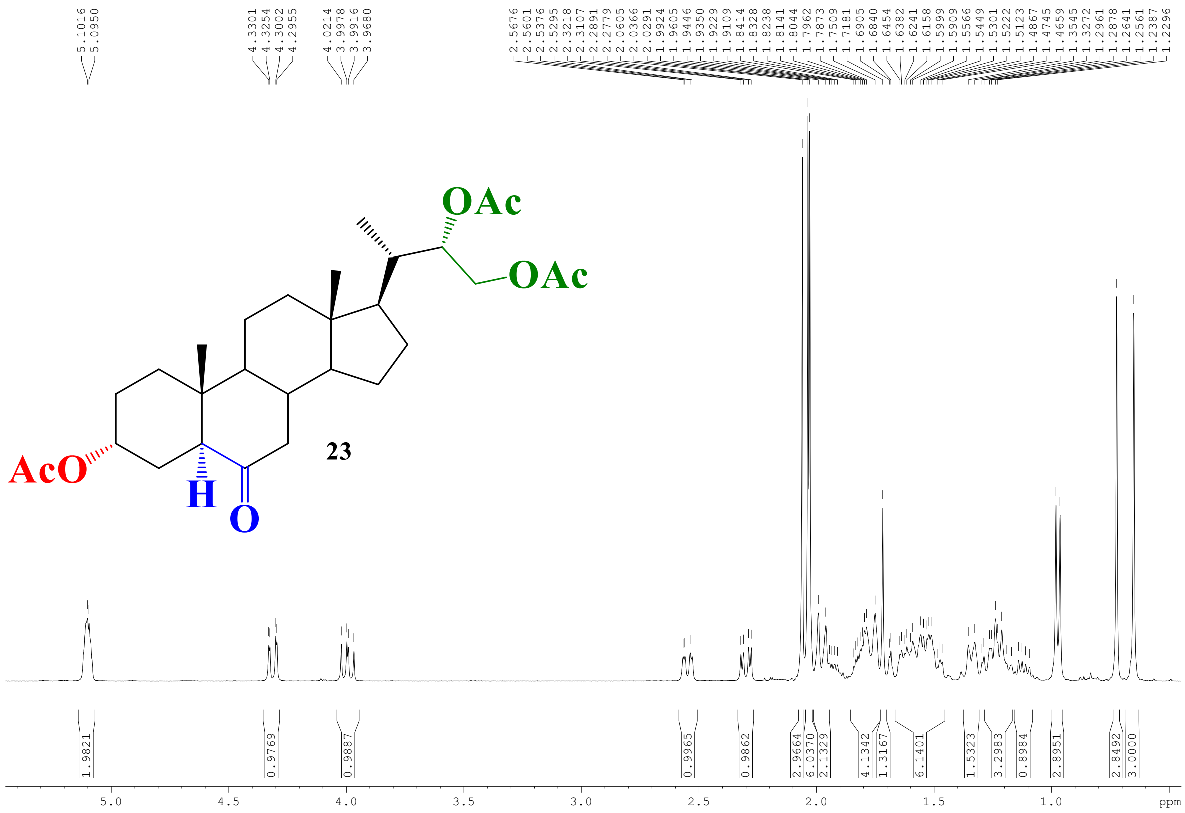

5.1016
5.0950
4.3301
4.3254
4.3002
4.2955
4.0214
3.9978
3.9916
3.9680
2.5676
2.5601
2.5376
2.5295
2.3218
2.3107
2.2891
2.2779
2.0605
2.0366
2.0291
1.9924
1.9605
1.9446
1.9350
1.9229
1.9109
1.8414
1.8328
1.8238
1.8141
1.8044
1.7962
1.7873
1.7509
1.7181
1.6905
1.6840
1.6454
1.6382
1.6241
1.6158
1.5999
1.5909
1.5566
1.5549
1.5301
1.5222
1.5123
1.4867
1.4745
1.4659
1.3545
1.3272
1.2961
1.2878
1.2641
1.2561
1.2387
1.2296
OAc
OAc
AcO
H
O
23
1.9821
0.9769
0.9887
0.9965
0.9862
2.9664
6.0370
2.1329
4.1342
1.3167
6.1401
1.5323
3.2983
0.8984
2.8951
2.8492
3.0000
5.0
4.5
4.0
3.5
3.0
2.5
2.0
1.5
1.0
ppm

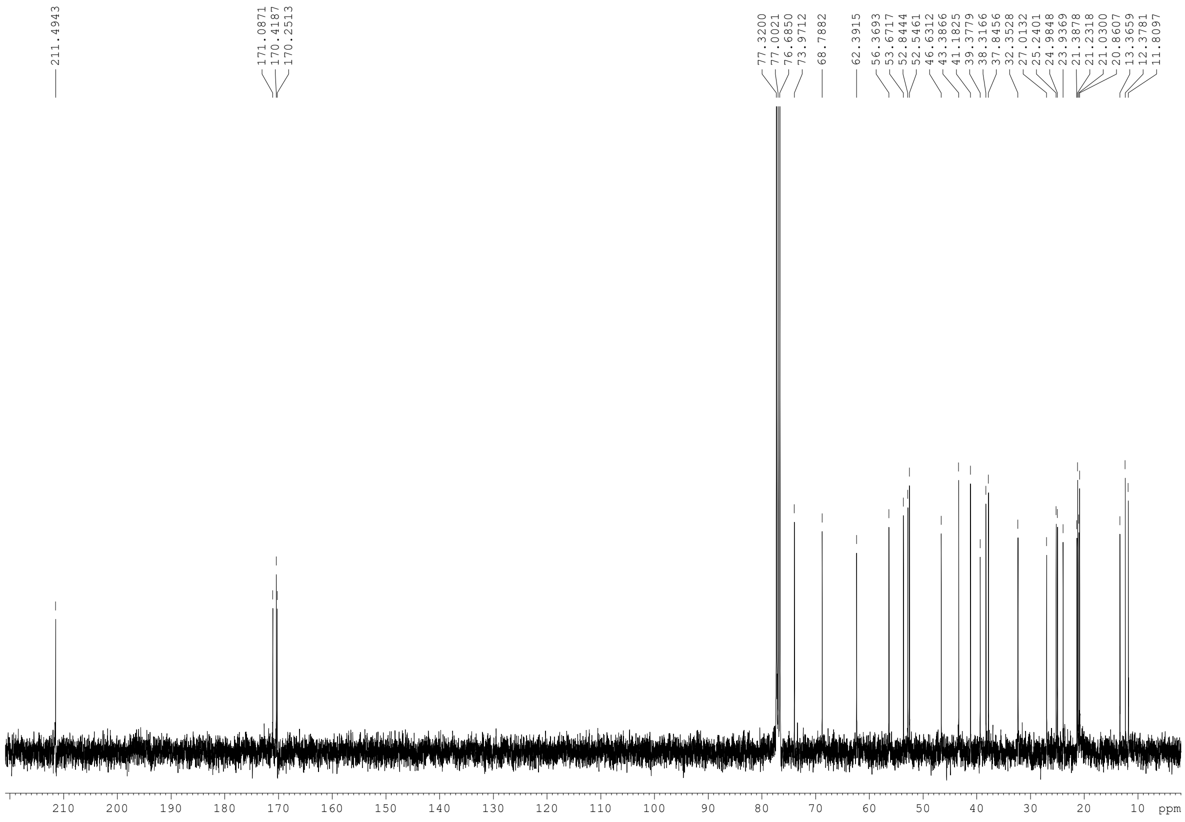

211.4943
171.0871
170.4187
170.2513
77.3200
77.0021
76.6850
73.9712
68.7882
62.3915
56.3693
53.6717
52.8444
52.5461
46.6312
43.3866
41.1825
39.3779
38.3166
37.8456
32.3528
27.0132
25.2401
24.9848
23.9369
21.3878
21.2318
21.0300
20.8607
13.3659
12.3781
11.8097
210
200
190
180
170
160
150
140
130
120
110
100
90
80
70
60
50
40
30
20
10
ppm

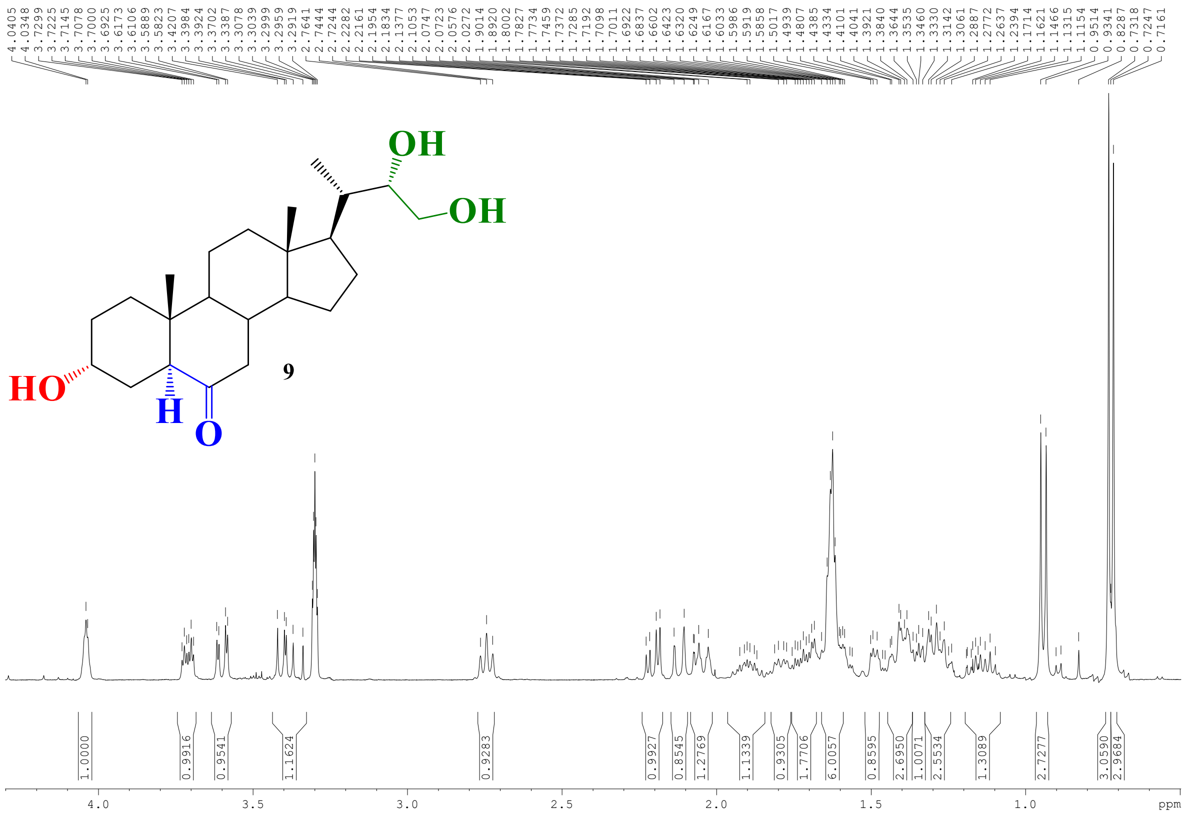

4.0405
4.0348
3.7299
3.7225
3.7145
3.7078
3.7000
3.6925
3.6173
3.6106
3.5889
3.5823
3.4207
3.3984
3.3924
3.3702
3.3387
3.3078
3.3039
3.2999
3.2959
3.2919
2.7641
2.7444
2.7244
2.2282
2.2161
2.1954
2.1834
2.1377
2.1053
2.0747
2.0723
2.0576
2.0272
1.9014
1.8920
1.8002
1.7827
1.7734
1.7459
1.7372
1.7285
1.7192
1.7098
1.7011
1.6922
1.6837
1.6602
1.6423
1.6320
1.6249
1.6167
1.6033
1.5986
1.5919
1.5858
1.5017
1.4939
1.4807
1.4385
1.4334
1.4101
1.4041
1.3921
1.3840
1.3644
1.3535
1.3460
1.3330
1.3142
1.3061
1.2887
1.2772
1.2637
1.2394
1.1714
1.1621
1.1466
1.1315
1.1154
0.9514
0.9341
0.8287
0.7318
0.7247
0.7161
OH
OH
HO
H
O
9
1.0000
0.9916
0.9541
1.1624
0.9283
0.9927
0.8545
1.2769
1.1339
0.9305
1.7706
6.0057
0.8595
2.6950
1.0071
2.5534
1.3089
2.7277
3.0590
2.9684
4.0
3.5
3.0
2.5
2.0
1.5
1.0
ppm

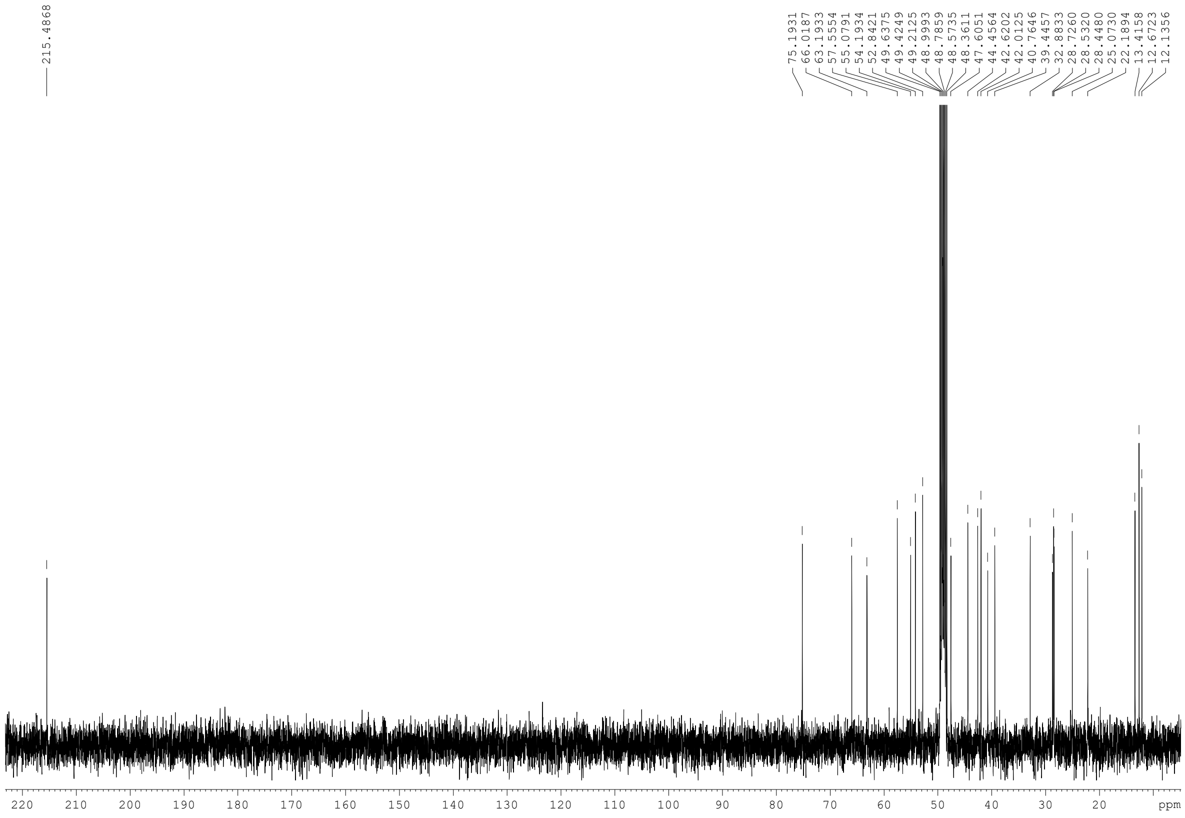

215.4868
75.1931
66.0187
63.1933
57.5554
55.0791
54.1934
52.8421
49.6375
49.4249
49.2125
48.9993
48.7859
48.5735
48.3611
47.6051
44.4564
42.6202
42.0125
40.7646
39.4457
32.8833
28.7260
28.5320
28.4480
25.0730
22.1894
13.4158
12.6723
12.1356
220
210
200
190
180
170
160
150
140
130
120
110
100
90
80
70
60
50
40
30
20
ppm

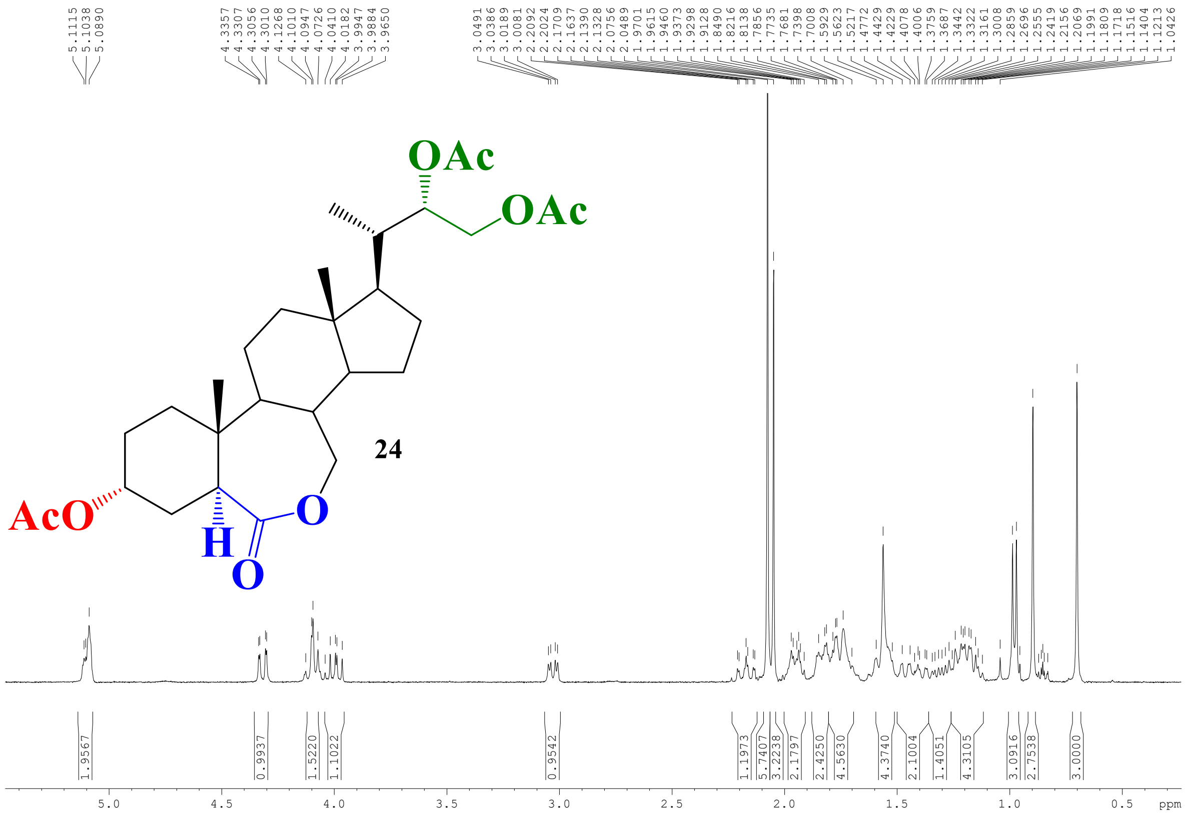
5.1115
5.1038
5.0890
4.3357
4.3307
4.3056
4.3010
4.1268
4.1010
4.0947
4.0726
4.0410
4.0182
3.9947
3.9884
3.9650
3.0491
3.0386
3.0189
3.0081
2.2092
2.2024
2.1709
2.1637
2.1390
2.1328
2.0756
2.0489
1.9701
1.9615
1.9460
1.9373
1.9298
1.9128
1.8490
1.8216
1.8138
1.7856
1.7735
1.7681
1.7398
1.7008
1.5929
1.5623
1.5217
1.4772
1.4429
1.4229
1.4078
1.4006
1.3759
1.3687
1.3442
1.3322
1.3161
1.3008
1.2859
1.2696
1.2555
1.2419
1.2156
1.2069
1.1991
1.1809
1.1718
1.1516
1.1404
1.1213
1.0426
OAc
OAc
24
AcO
H
O
O
1.9567
0.9937
1.5220
1.1022
0.9542
1.1973
5.7407
3.2238
2.1797
2.4250
4.5630
4.3740
2.1004
1.4051
4.3105
3.0916
2.7538
3.0000
5.0
4.5
4.0
3.5
3.0
2.5
2.0
1.5
1.0
0.5
ppm

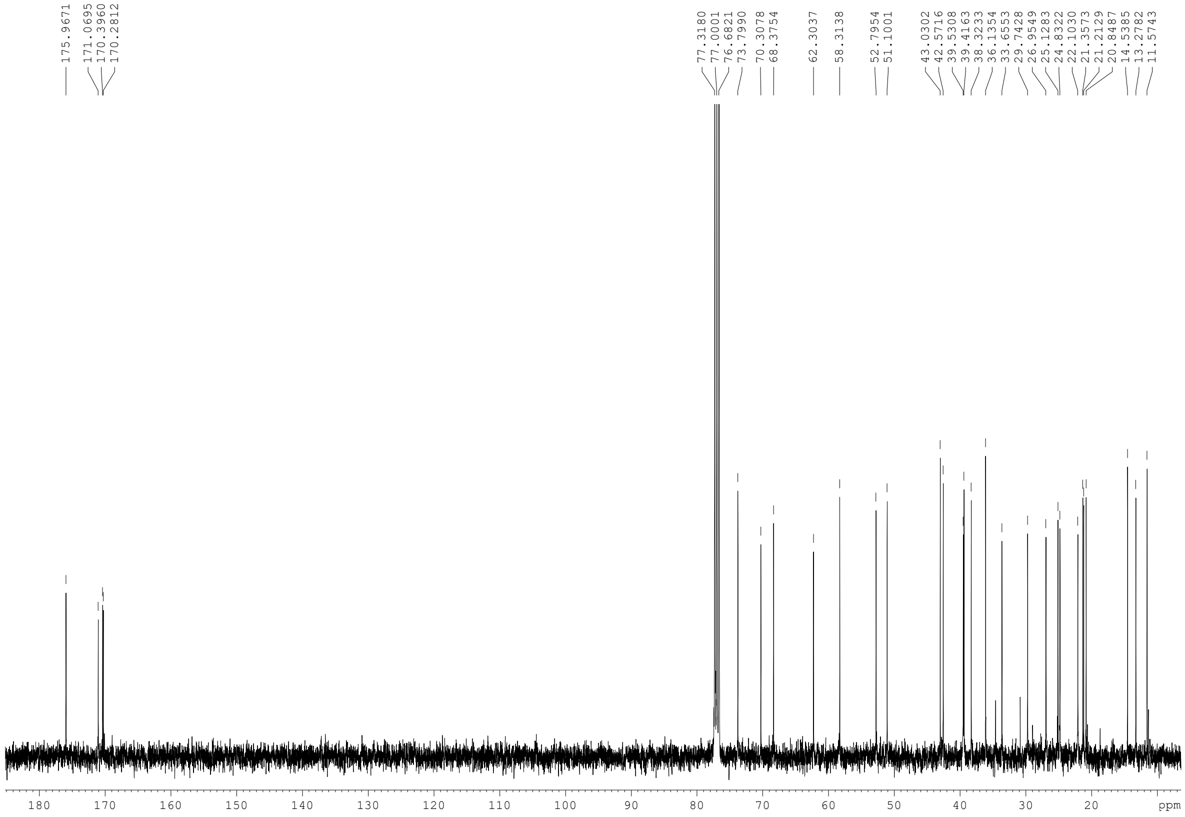
175.9671
171.0695
170.3960
170.2812
77.3180
77.0001
76.6821
73.7990
70.3078
68.3754
62.3037
58.3138
52.7954
51.1001
43.0302
42.5716
39.5308
39.4163
38.3233
36.1354
33.6553
29.7428
26.9549
25.1283
24.8322
22.1030
21.3573
21.2129
20.8487
14.5385
13.2782
11.5743
180
170
160
150
140
130
120
110
100
90
80
70
60
50
40
30
20
ppm

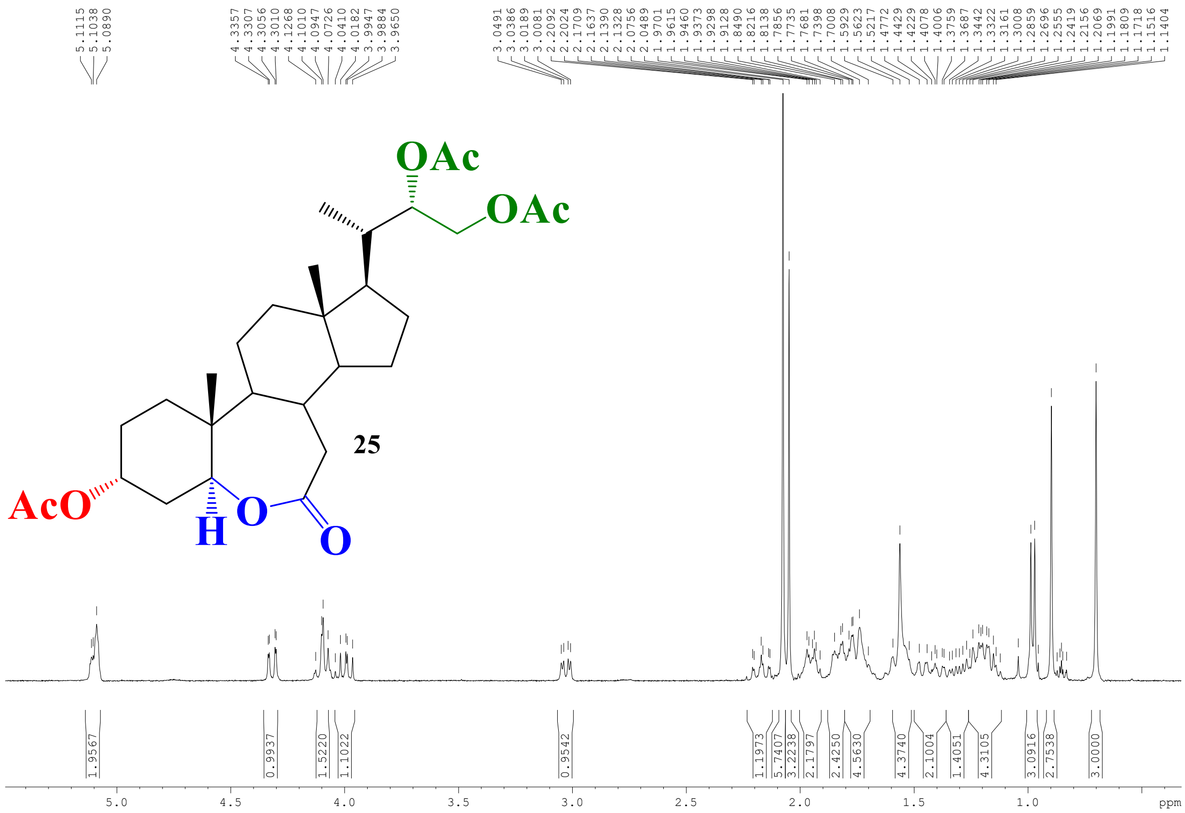
5.1115
5.1038
5.0890
4.3357
4.3307
4.3056
4.3010
4.1268
4.1010
4.0947
4.0726
4.0410
4.0182
3.9947
3.9884
3.9650
3.0491
3.0386
3.0189
3.0081
2.2092
2.2024
2.1709
2.1637
2.1390
2.1328
2.0756
2.0489
1.9701
1.9615
1.9460
1.9373
1.9298
1.9128
1.8490
1.8216
1.8138
1.7856
1.7735
1.7681
1.7398
1.7008
1.5929
1.5623
1.5217
1.4772
1.4429
1.4229
1.4078
1.4006
1.3759
1.3687
1.3442
1.3322
1.3161
1.3008
1.2859
1.2696
1.2555
1.2419
1.2156
1.2069
1.1991
1.1809
1.1718
1.1516
1.1404
OAc
OAc
AcO
H
O
O
25
1.9567
0.9937
1.5220
1.1022
0.9542
1.1973
5.7407
3.2238
2.1797
2.4250
4.5630
4.3740
2.1004
1.4051
4.3105
3.0916
2.7538
3.0000
5.0
4.5
4.0
3.5
3.0
2.5
2.0
1.5
1.0
ppm

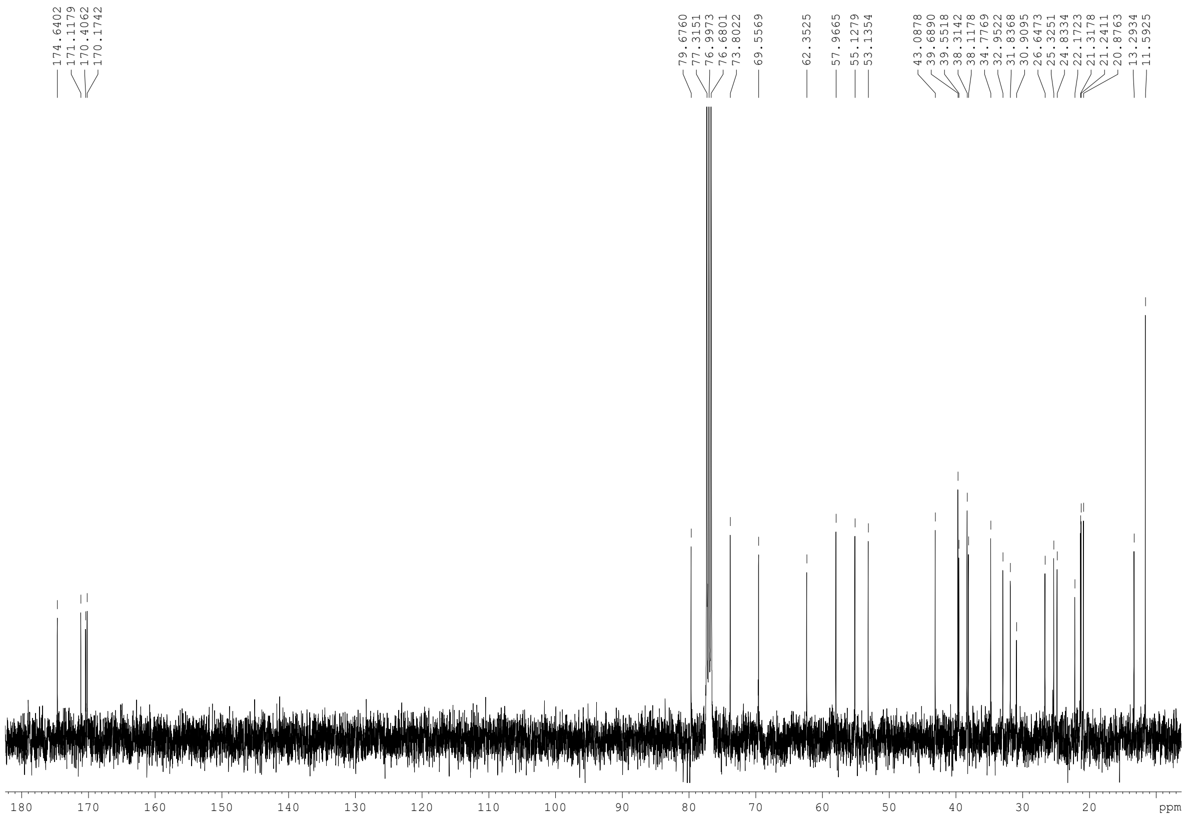
174.6402
171.1179
170.4062
170.1742
79.6760
77.3151
76.9973
76.6801
73.8022
69.5569
62.3525
57.9665
55.1279
53.1354
43.0878
39.6890
39.5518
38.3142
38.1178
34.7769
32.9522
31.8368
30.9095
26.6473
25.3251
24.8334
22.1723
21.3178
21.2411
20.8763
13.2934
11.5925
180
170
160
150
140
130
120
110
100
90
80
70
60
50
40
30
20
ppm

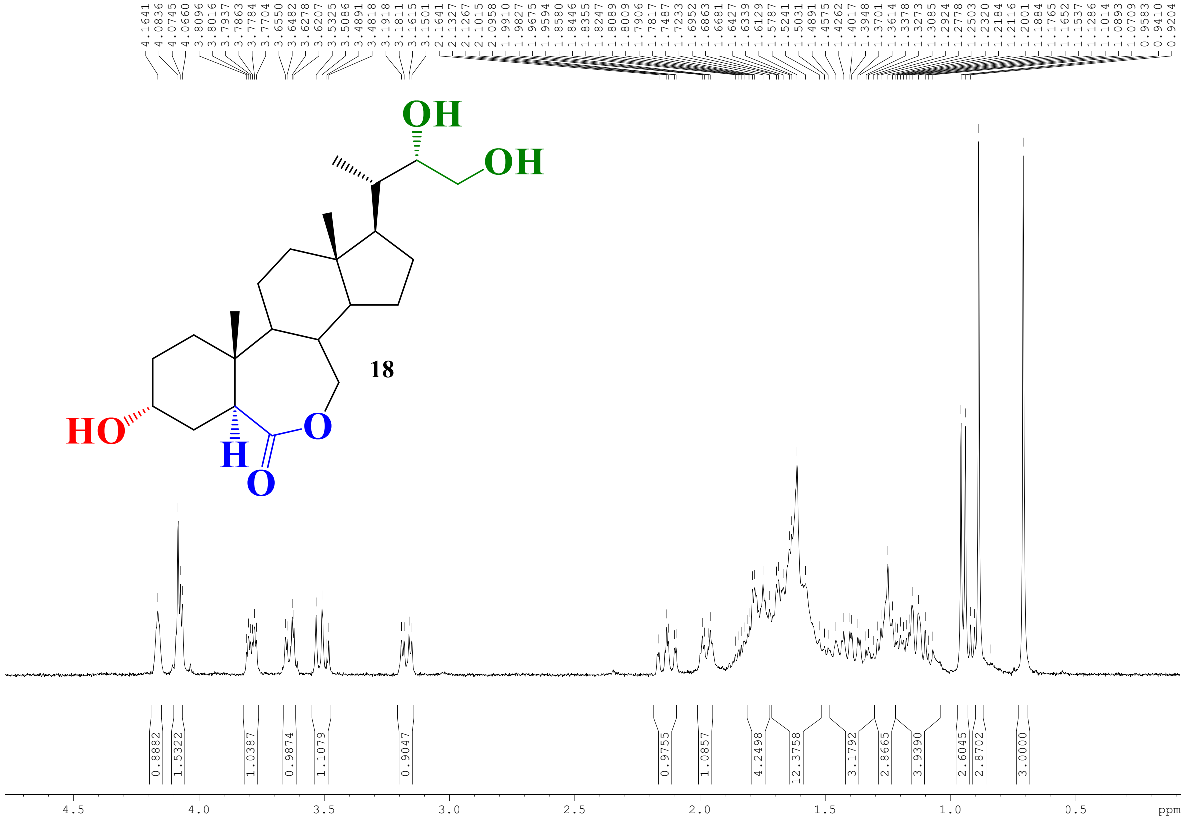
4.1641 4.0836 4.0745 4.0660 3.8096 3.8016 3.7937 3.7863 3.7784 3.7704 3.6550 3.6482 3.6278 3.6207 3.5325 3.5086 3.4891 3.4818 3.1918 3.1811 3.1615 3.1501 2.1641 2.1327 2.1267 2.1015 2.0958 1.9910 1.9827 1.9675 1.9594 1.8580 1.8446 1.8355 1.8247 1.8089 1.8009 1.7906 1.7817 1.7487 1.7233 1.6952 1.6863 1.6681 1.6427 1.6339 1.6129 1.5787 1.5241 1.5031 1.4891 1.4575 1.4262 1.4017 1.3948 1.3701 1.3614 1.3378 1.3273 1.3085 1.2924 1.2778 1.2503 1.2320 1.2184 1.2116 1.2001 1.1884 1.1765 1.1652 1.1537 1.1286 1.1014 1.0893 1.0709 0.9583 0.9410 0.9204
OH
OH
HO
H
O
O
18
0.8882
1.5322
1.0387
0.9874
1.1079
0.9047
0.9755
1.0857
4.2498
12.3758
3.1792
2.8665
3.9390
2.6045
2.8702
3.0000
4.5
4.0
3.5
3.0
2.5
2.0
1.5
1.0
0.5
ppm

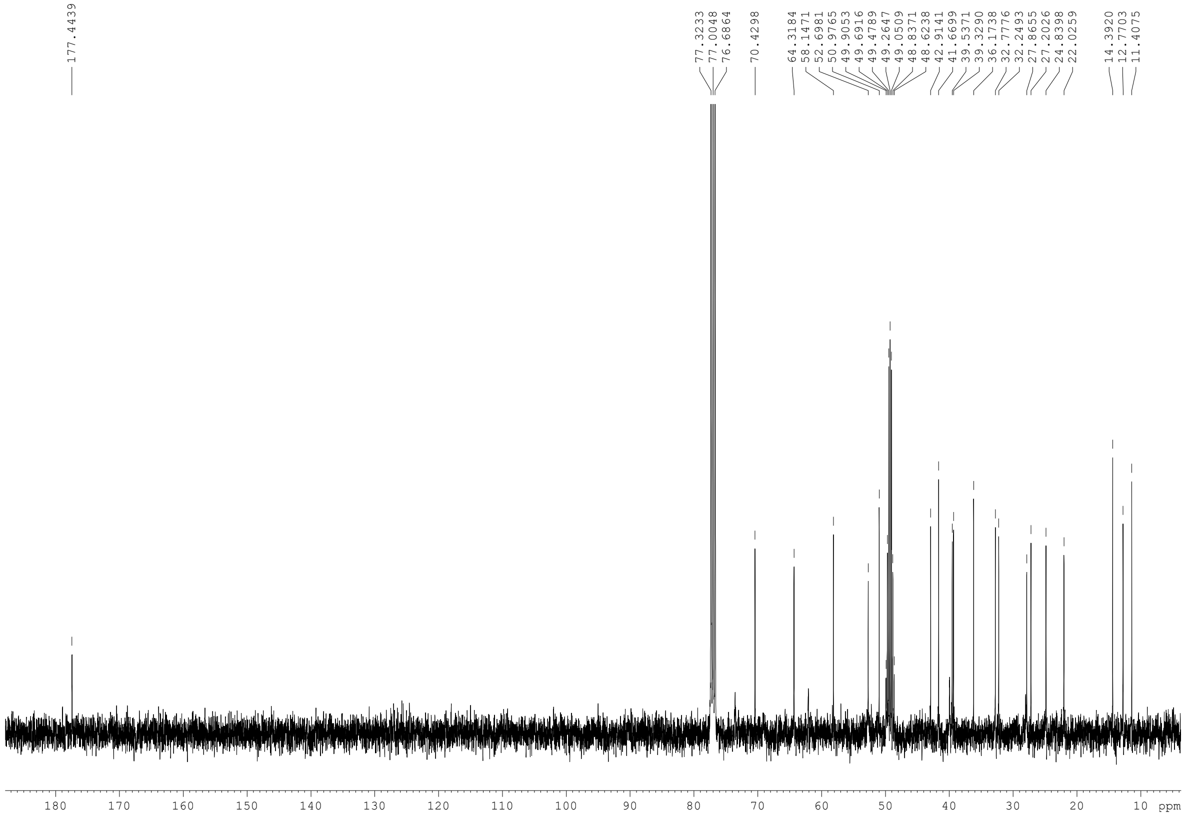
177.4439
77.3233 77.0048 76.6864
70.4298
64.3184 58.1471 52.6981 50.9765 49.9053 49.6916 49.4789 49.2647 49.0509 48.8371 48.6238 42.9141 41.6699 39.5371 39.3290 36.1738 32.7776 32.2493 27.8655 27.2026 24.8398 22.0259
14.3920 12.7703 11.4075
180
170
160
150
140
130
120
110
100
90
80
70
60
50
40
30
20
10
ppm

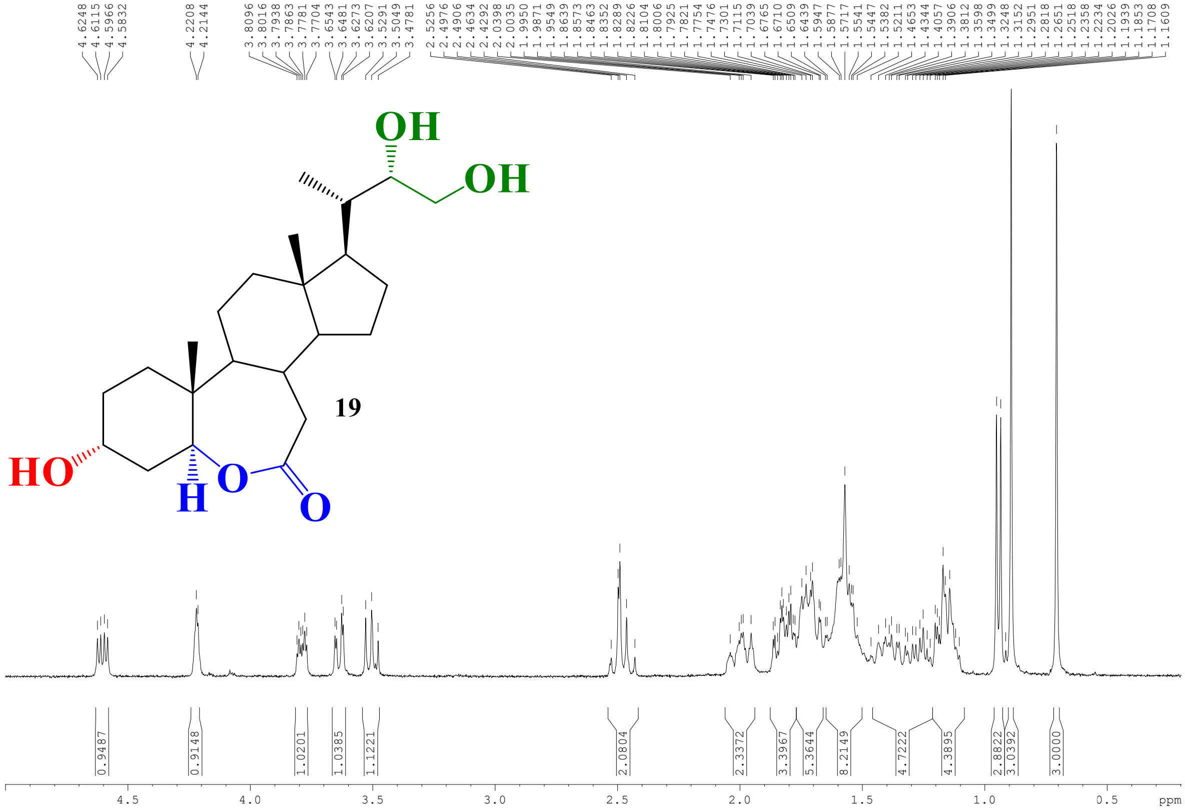

OH
OH
HO
H
O
O
19
4.6248 4.6115 4.5966 4.5832 4.2208 4.2144 3.8096 3.8016 3.7938 3.7863 3.7781 3.7704 3.6543 3.6481 3.6273 3.6207 3.5291 3.5049 3.4781 2.5256 2.4976 2.4906 2.4634 2.4292 2.0398 2.0035 1.9950 1.9871 1.9549 1.8639 1.8573 1.8463 1.8352 1.8289 1.8226 1.8104 1.8006 1.7925 1.7821 1.7754 1.7476 1.7301 1.7115 1.7039 1.6765 1.6710 1.6509 1.6439 1.5947 1.5877 1.5717 1.5541 1.5447 1.5382 1.5211 1.4653 1.4344 1.4057 1.3906 1.3812 1.3598 1.3499 1.3248 1.3152 1.2951 1.2818 1.2651 1.2518 1.2358 1.2234 1.2026 1.1939 1.1853 1.1708 1.1609
0.9487 0.9148 1.0201 1.0385 1.1221 2.0804 2.3372 3.3967 5.3644 8.2149 4.7222 4.3895 2.8822 3.0392 3.0000
4.5 4.0 3.5 3.0 2.5 2.0 1.5 1.0 0.5 ppm

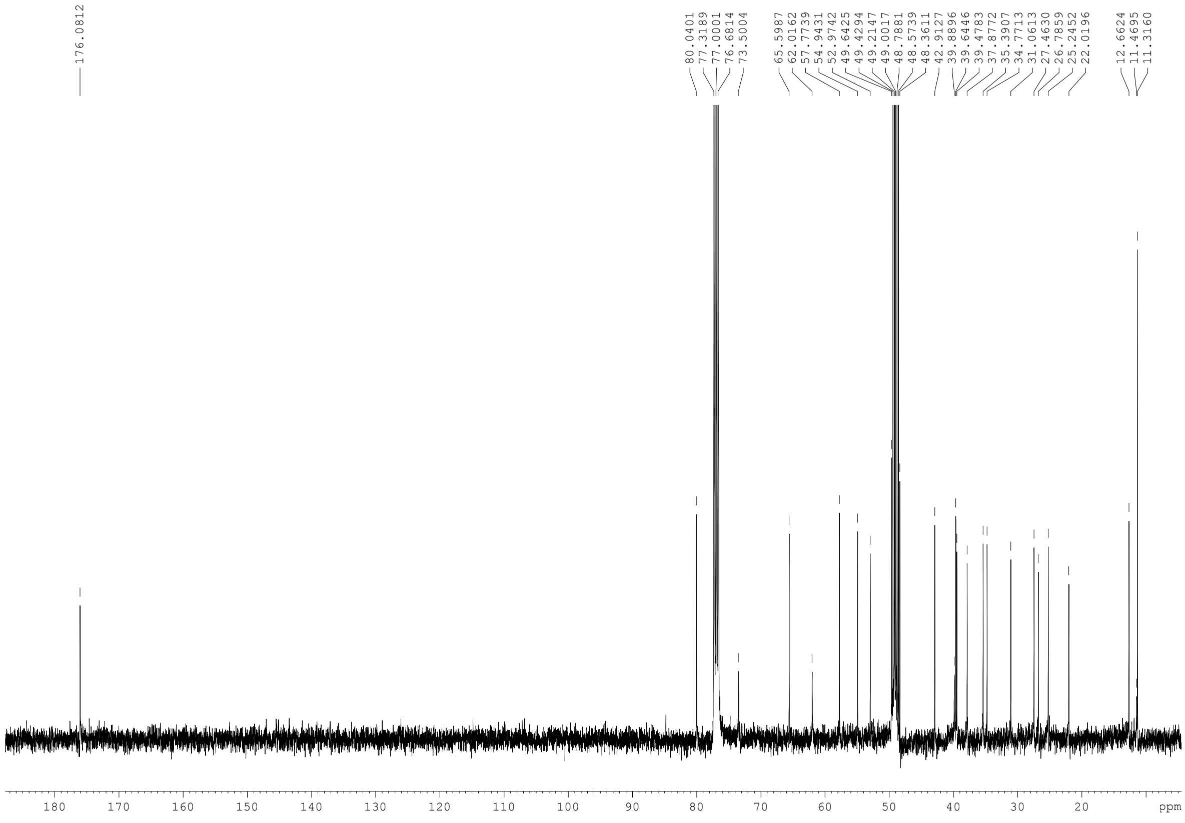

176.0812
80.0401 77.3189 77.0001 76.6814 73.5004
65.5987 62.0162 57.7739 54.9431 52.9742 49.6425 49.4294 49.2147 49.0017 48.7881 48.5739 48.3611 42.9127 39.8896 39.6446 39.4783 37.8772 35.3907 34.7713 31.0613 27.4630 26.7859 25.2452 22.0196
12.6624 11.4695 11.3160
180 170 160 150 140 130 120 110 100 90 80 70 60 50 40 30 20 ppm

S3. Copies of HRMS

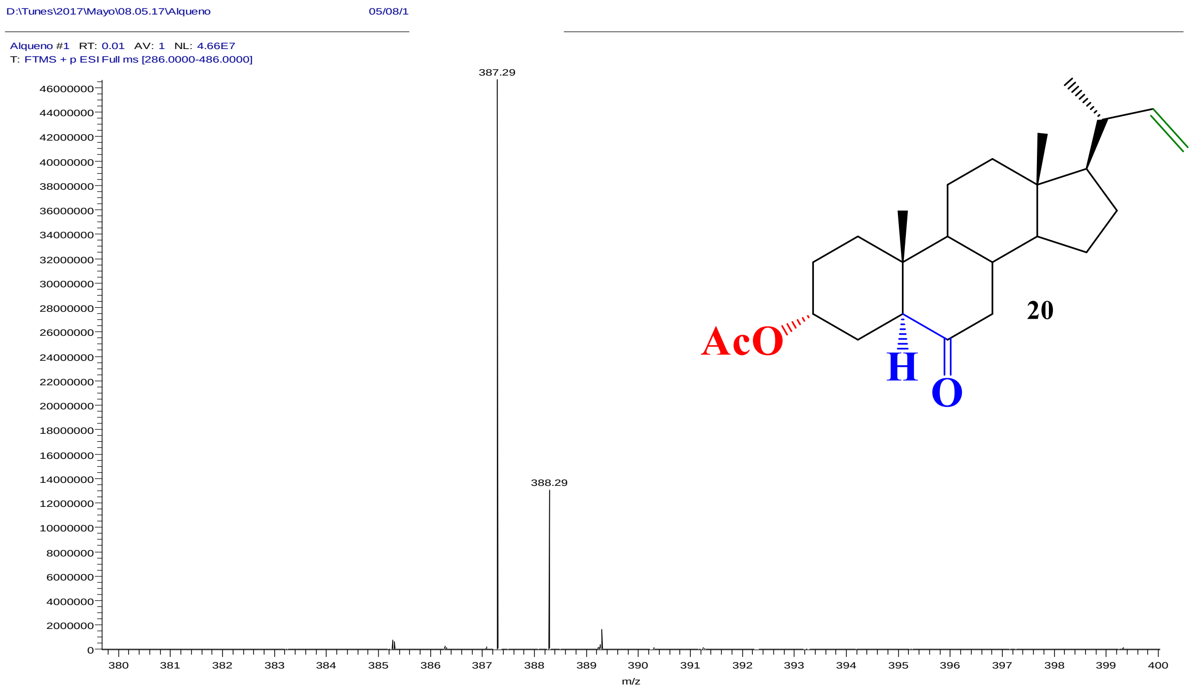

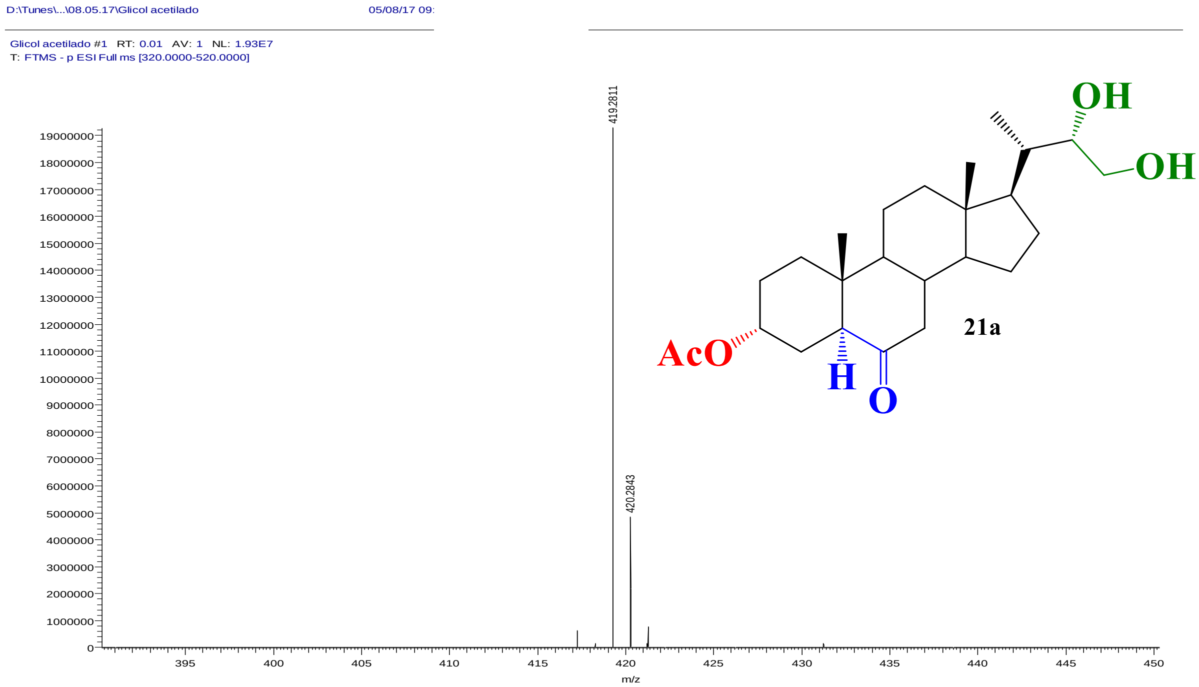

D:\Tunes\2017\Mayo\08.05.17\Benzoilado 05/08/17 09:

Benzoilado #1 RT: 0.01 AV: 1 NL: 2.57E7
T: FTMS + p ESI Full ms [424.0000-624.0000]

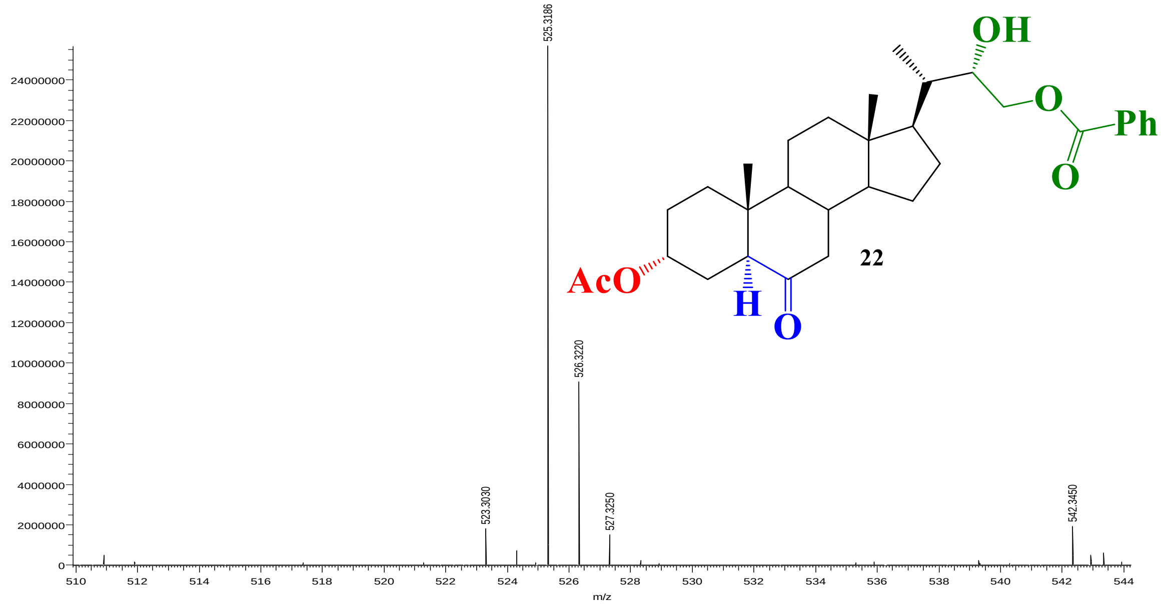

D:\Tunes\2017\Mayo\08.05.17\Triacetilado 05/08/17 10:

Triacetilado #1 RT: 0.01 AV: 1 NL: 7.47E6
T: FTMS + p ESI Full ms [405.0000-605.0000]

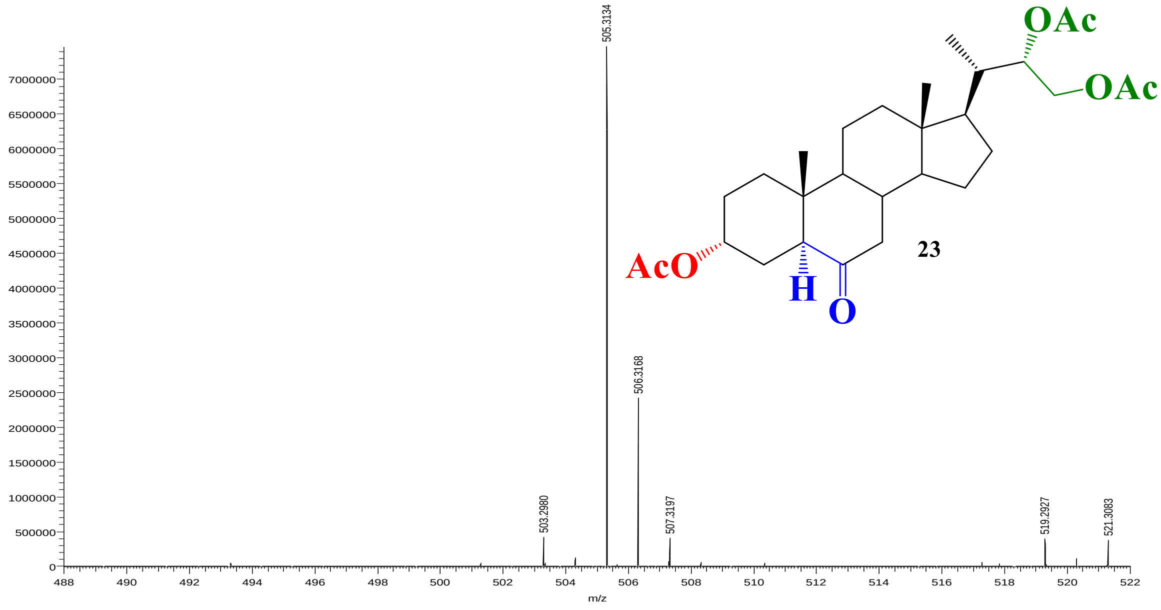

D:\Tunes\2017\Mayo\08.05.17\Triol 05/08/17 09:

Triol #3 RT: 0.03 AV: 1 NL: 3.37E6
T: FTMS + p ESI Full ms [278.0000-478.0000]

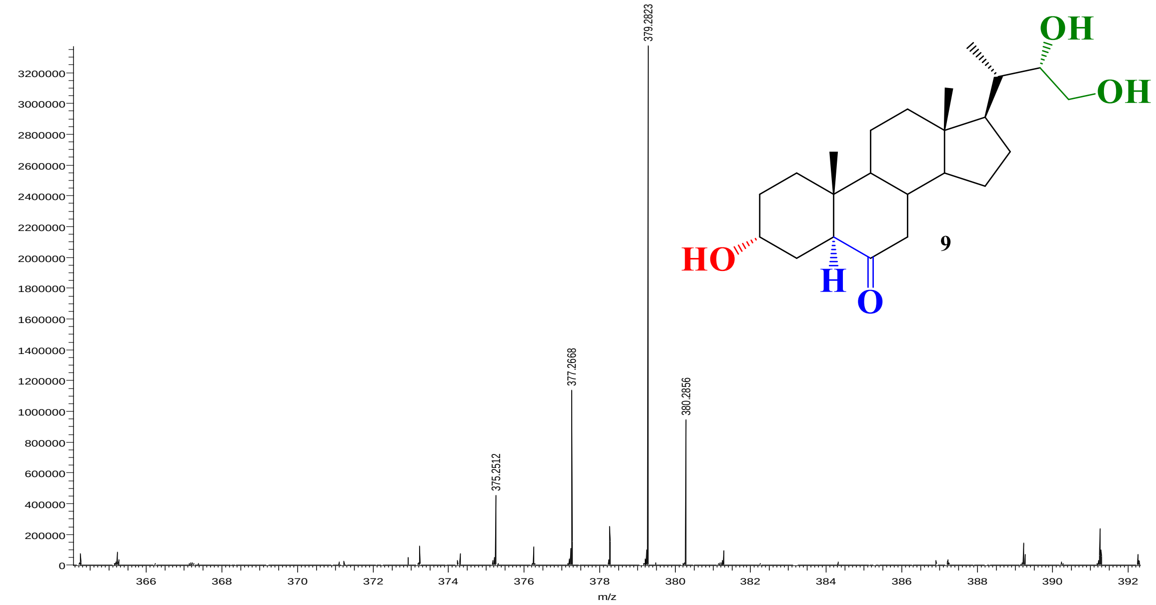

D:\Tunes\...\Triacetilado lactona 7 05/08/17 09:

Triacetilado lactona 7 #1 RT: 0.01 AV: 1 NL: 4.95E7
T: FTMS + p ESI Full ms [421.0000-621.0000]

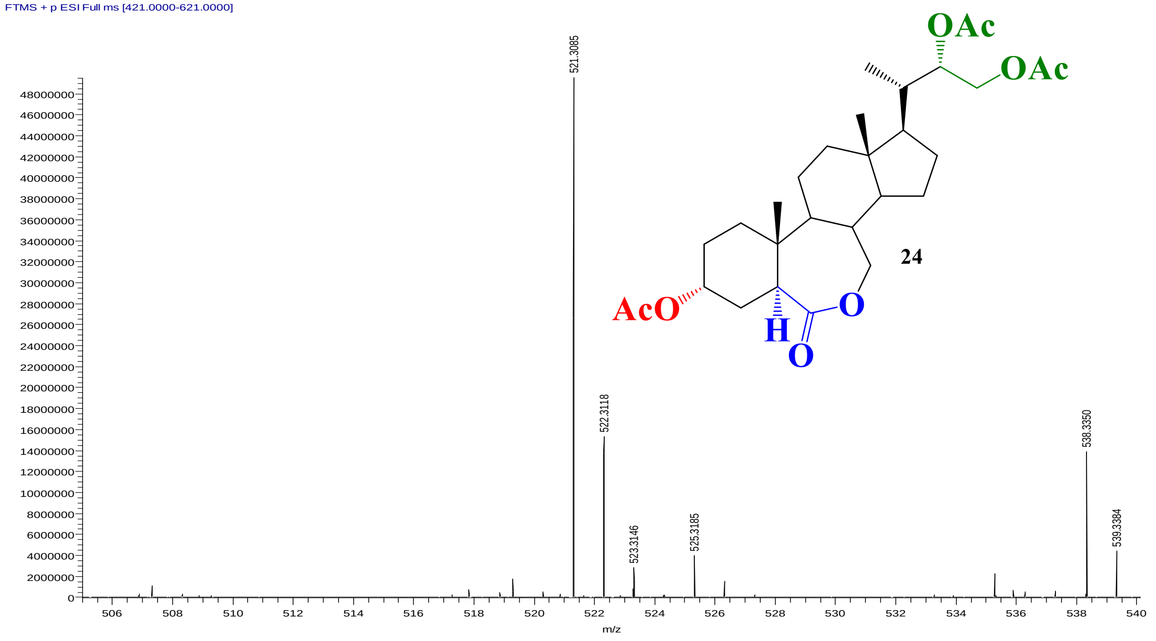

D:\Tunes\...\Triacetilado lactona-6 05/08/17 09:

Triacetilado lactona-6 #1 RT: 0.01 AV: 1 NL: 6.57E6
T: FTMS + p ESI Full ms [420.0000-620.0000]

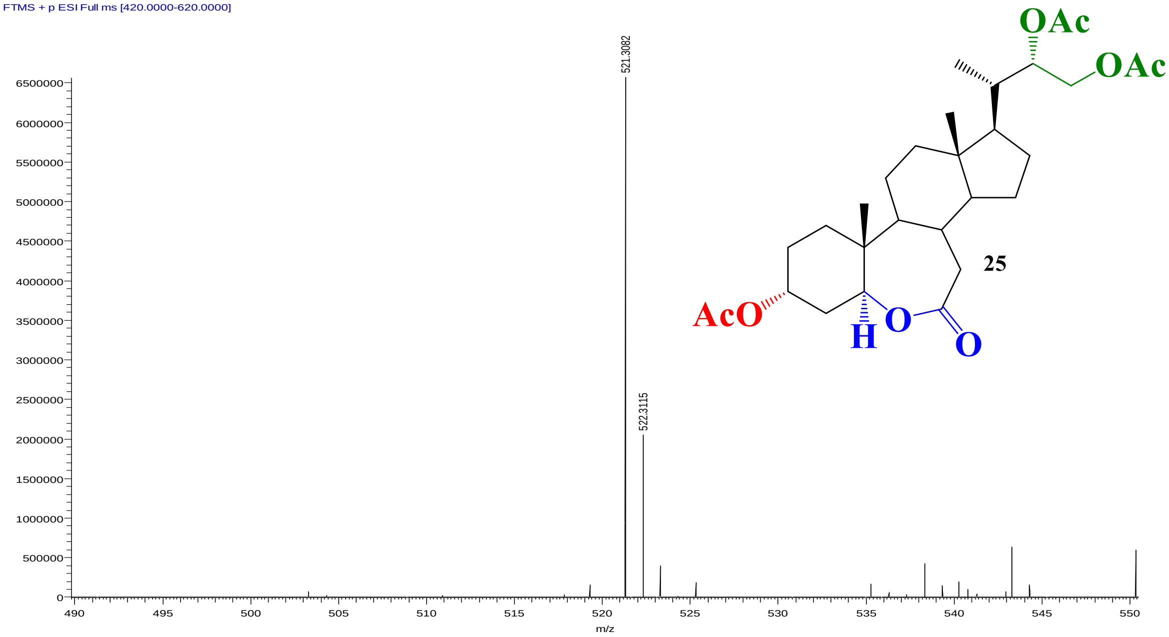

25

D:\Tunes\...\Mayo\08.05.17\7-Oxo lactona 05/08/17 09:

7-Oxo lactona #1 RT: 0.01 AV: 1 NL: 2.79E7
T: FTMS + p ESI Full ms [294.0000-494.0000]

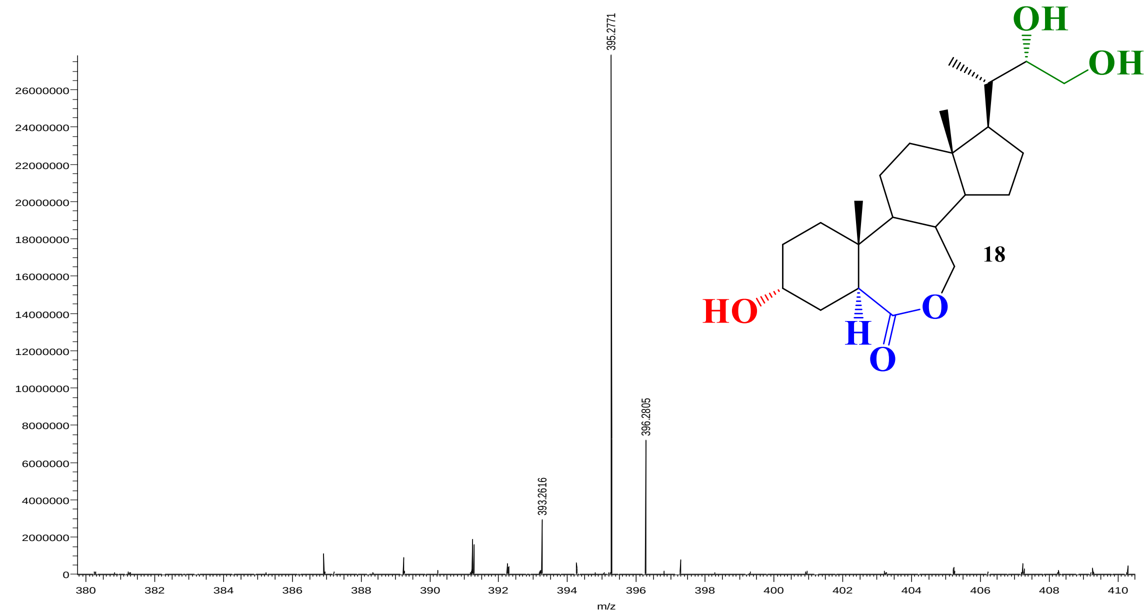

18

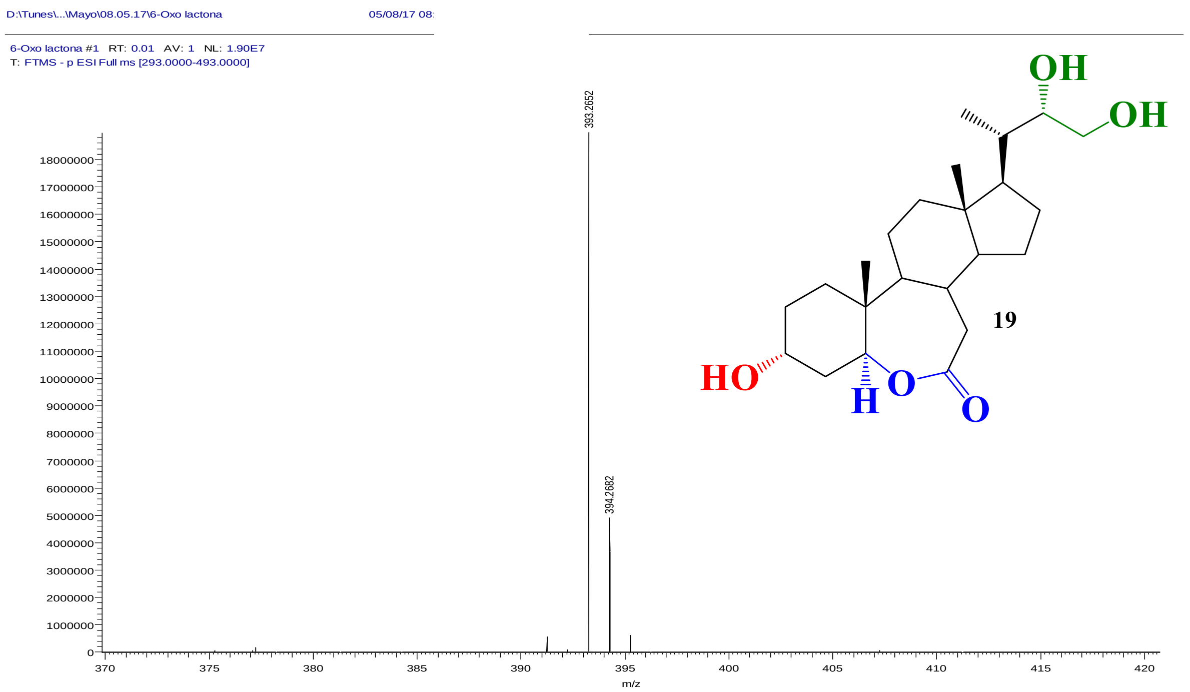
D:\Tunes\...\Mayo\08.05.17\6-Oxo lactona
05/08/17 08:
6-Oxo lactona #1 RT: 0.01 AV: 1 NL: 1.90E7
T: FTMS - p ESI Full ms [293.0000-493.0000]
393.2652
394.2682
m/z
OH
OH
HO
H
O
O
19
